# Probabilities of two alleles being identity by state at unobserved loci predicted by observed loci in cattle populations

**DOI:** 10.1038/s41598-026-37530-x

**Published:** 2026-02-05

**Authors:** Rintaro Nagai, Takeshi Honda, Masahiro Satoh, Yoshinobu Uemoto

**Affiliations:** 1 Graduate School of Agricultural Science, Tohoku University, Sendai, 980-8572 Miyagi Japan; 2 Food Resources Education and Research Center, Kobe University, Kasai, 675-2103 Hyogo Japan

**Keywords:** Additive relationship coefficient, Genetic diversity, Identity-by-descent, Inbreeding coefficient, Simulated and real cattle populations, Animal breeding, Genetic markers, Inbreeding

## Abstract

This study aimed to investigate the prediction accuracy of the probability that alleles at unobserved loci are identity-by-state (IBS) using genome-based measures based on observed single nucleotide polymorphisms (SNPs). We performed a simulation analysis assumed to represent a cattle population with simulated and real SNP genotypes. The genome-based measures were based on the inbreeding coefficients in an individual and the additive relationship coefficients between two individuals. Reference values were defined as the probability that the alleles at unobserved SNPs were IBS. Reference values were predicted using both pedigree-based and genome-based measures with tens of thousands of SNPs. Prediction accuracy was calculated as the correlation coefficient between reference and predicted values. Our results showed that the inbreeding and additive relationship coefficients based on SNP-by-SNP with an allele frequency fixed at 0.5 and the coefficients based on the homozygous-segment with homozygous by descent and with run of homozygosity > 4 Mbp long demonstrated consistent high prediction accuracy in both simulated and real cattle populations. Our results also showed that the correlation coefficients of these measures were higher than those of pedigree-based measures. Our results indicate that genome-based measures utilizing observed SNPs can offer a more accurate prediction of IBS relationships at unobserved loci than pedigree-based measures in cattle populations.

**Supplementary Information:**

The online version contains supplementary material available at 10.1038/s41598-026-37530-x.

## Introduction

Maintaining genetic diversity in cattle breeds has been a long-standing issue, as evidenced by reports of the loss of genetic diversity in many cattle breeds^1–5^. Genetic diversity allows for selection flexibility to enhance the productivity of both existing and new economically important traits in the current population, as well as for adaptation to ensure food security and resilience in the context of future environmental changes in the future population^6,7^. Furthermore, reduced genetic diversity results in an increase of the homozygosity in the population. This may lead to an elevated frequency of recessive harmful alleles, which could subsequently impair individual performance (i.e., inbreeding depression^8^), with regard to fitness traits such as production, health, and reproductive traits in cattle^9–12^.

Genetic diversity in cattle breeds has traditionally been managed by controlling inbreeding rates using inbreeding coefficients and additive relationship coefficients derived from pedigree information^8^. The inbreeding coefficient is defined as the probability that two homologous alleles at neutral loci that are unlinked to any locus under selection are identity-by-descent (IBD) in an individual^8,13^. The additive relationship coefficient, also referred to as the numerator relationship coefficient, is based on the concept of coancestry coefficients, which are defined as the probability that two homologous alleles at neutral loci are IBD between two individuals with a common ancestor. The additive relationship coefficient between two individuals is twice the coancestry coefficient^8^. This approach was adopted to avoid mating between animals with a common ancestor. However, pedigree-based measures may not accurately measure IBD because they may be influenced by biases associated with the definition of the base population^14^. If pedigree information contains errors in its recording, or if the depth of the pedigree is insufficient, it will not be possible to ascertain the precise base population^15^.

Genomic information offers new possibilities for monitoring the level of inbreeding. Genome-based measures have the potential to provide more accurate estimation of realized genomic inbreeding, and could be used in breeding strategies to control inbreeding more effectively than pedigree information^14,16,17^. Many types of genome-based inbreeding coefficients and additive relationship coefficients have been proposed to predict the probability of IBD at the genome level without pedigree information by utilizing high- or middle-density single nucleotide polymorphism (SNP) arrays^18^. Genome-based measures reflect the actual proportion of the genome shared by individuals and offer a more accurate representation than pedigree-based measures, which are limited to the expected fractions of genomic IBD. The use of these measures has facilitated the monitoring of genetic diversity in cattle breeds.

Genetic diversity monitoring using genome-based measures is focused on two points: preserving the genetic variability of causal variants at unknown loci that are relevant to future breeding goals and controlling the increased homozygosity of recessive harmful alleles at unknown loci. Consequently, the use of genome-based measures based on SNPs at observed loci involves predicting the probability that alleles at unobserved loci are identity-by-state (IBS) in an individual and between two individuals^19,20^. IBS is that two homologous alleles at a single locus are identical merely by state, irrespective of whether they were inherited from a recent common ancestor^19^. Although several studies have compared genome-based measures with pedigree-based coefficients or with simulated IBD/IBS values^17,21,22^, few have explicitly evaluated their predictive accuracy for IBS at unobserved loci, particularly when combining simulated and real cattle populations. Therefore, the objective of this study was to investigate the accuracy of genome-based measures with observed SNPs for predicting the probability that alleles at unobserved loci are IBS in both simulated and real cattle populations. The genome-based measures were based on the inbreeding coefficients in an individual and the additive relationship coefficients between two individuals. We performed a simulation analysis based on genotypes assumed to represent a cattle population, and real genotypes reflecting the extent of linkage disequilibrium (LD) in Japanese Black cattle. In the simulated cattle population, the differences in prediction accuracy between genome- and pedigree-based measures were compared.

## Materials and Methods

### Simulated cattle population

**Fig. 1:**
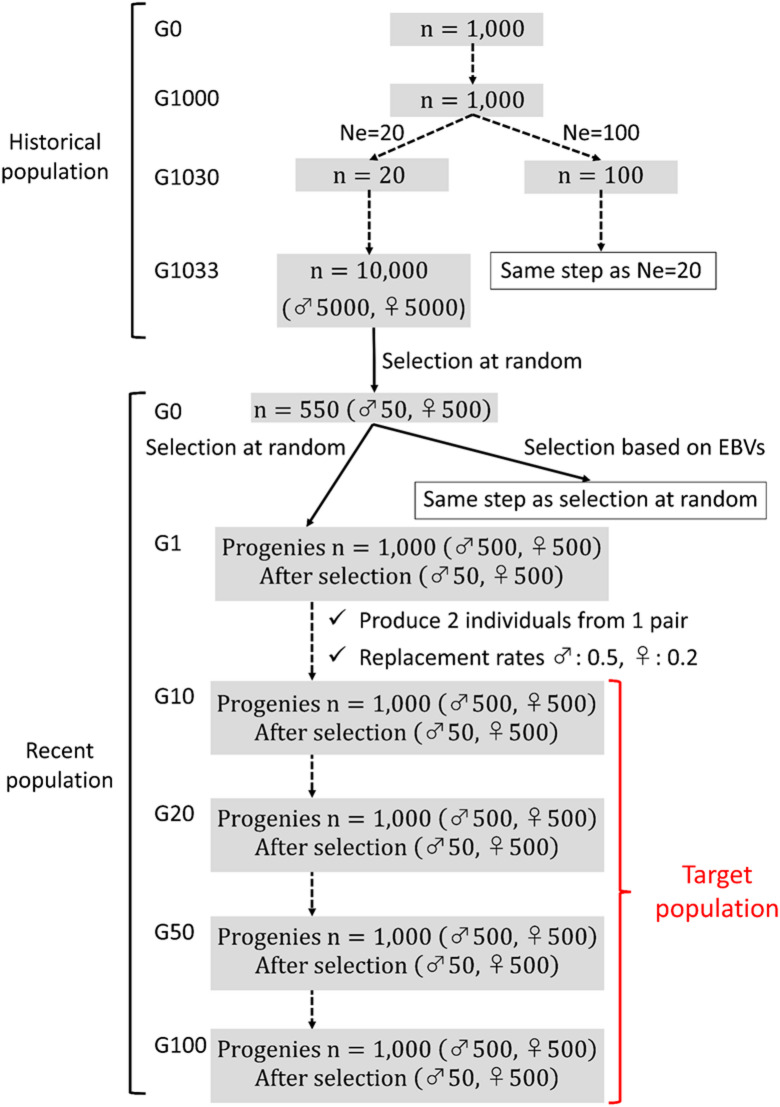
Schematic illustration of the simulation.

Populations were simulated based on the forward-in-time process^23^ using the QMSim software^24^. A schematic of the simulation process is shown in Fig. 1. First, a historical population was simulated to create mutation drift equilibrium and LD, which were the same as those described by Takeda et al.^25^. Briefly, the size of the historical population began with 1,000 individuals and the historical population was generated as generation zero (G0) to 1,000 (G1000), with a constant size of 1,000. Two different populations with different effective population size (*N*_*e*_) were generated with a *N*_*e*_ of 20 and 100^26^, which mimicked the recent *N*_*e*_ of Japanese Black cattle population^1^ and other cattle breed populations such as Holstein cattle^3,27^ and Japanese Shorthorn cattle^28^, respectively. Thus, the number of individuals gradually decreased from 1000 to 20 (for *N*_*e*_ =20) or 100 (for *N*_*e*_ =100) from generations 1001 to 1030. This was then expanded to 10,000 individuals after three generations, resulting in 5,000 males and 5000 females in the final generation. In each population, 50 males and 500 females were randomly selected from the last historical generation as founders of the recent population. Different patterns of LD decay between *N*_*e*_ =20 and *N*_*e*_ =100 were obtained (Supplementary Fig. S1), similar to those described by Takeda et al.^25^. The simulated population with *N*_*e*_ =20 had higher LD coefficient (r^2^) values^29^ than those with *N*_*e*_ =100 for all distances between the two loci.

Second, four different types of recent populations were simulated with varying *N*_*e*_ (*N*_*e*_ =20 or *N*_*e*_ =100) and selection criteria (selection at random or selection based on estimated breeding values (EBVs)) over 100 overlapping generations with a constant progeny size per generation (*n* = 1000). Each mating produced two progenies with equal changes in being male or female, and 1,000 progenies were obtained in each generation. Next, 50 male and 500 female progenies were then selected with replacement rate of 0.5 for males and 0.2 for females. The generated recent population was then used to evaluate the accuracy of the genome-based measures. The main parameters of the recent population are summarized in Table 1.

**Table 1:** Main parameters for the recent population using QMSim software.

Parameter	Value
Effective population size	20, 100
Number of generations	100 overlapping generations
Progeny size per generations	1,000
Number of breeding males	50
Number of breeding females	500
Number of females per male	10
Number of progeny per female	2
Replacement rate of selection	0.5 in males, 0.2 in females
Selection criteria	Random, higher EBV
Heritability	0.3
Number of chromosomes	29
Number of markers	60,000
Number of QTL	500
Number of replicates	100

For the genomic structure, the simulated genome consisted of 29 autosomal chromosomes and had a total length of 24.71 Morgans with a length similar to the bovine autosomes based on ARS-UCD 1.2 reference sequence assembly (https://www.ncbi.nlm.nih.gov/datasets/genome/GCF_002263795.1/). The details of the simulated genomes are presented in Supplementary Table S1. In the historical population, each chromosome contained twice as many SNPs as the Illumina BovineSNP50v2 BeadChip (Illumina Inc., San Diego, CA, USA) and 70 quantitative trait loci (QTL). The SNPs and QTL were biallelic and uniformly distributed along the chromosomes. The initial minor allele frequency (MAF) of SNPs and QTL was set to 0.5, and the mutation rates of SNPs and QTL were set to $\:2.5\times\:{10}^{-5}$. In the recent population, a total of 60,000 SNPs and 500 QTL with MAF ≥ 0.05 were randomly selected from all chromosomes in the founders. The QTL effects were sampled from a gamma distribution with a shape parameter of 0.4 and the scale parameter was determined internally for the simulated genetic variance. Simulated phenotypes with a set value of phenotypic variance (1.0) were generated with heritability (h^2^ = 0.30) explained only by the simulated QTL. All individuals had phenotypes that were used for selection based on EBVs predicted by the best linear unbiased prediction (BLUP). One hundred replicates of historical and recent populations were simulated based on the genomic structure for each condition.

In the simulated population, the target population was defined as generations 10 (G10), 20 (G20), 50 (G50), and 100 (G100) of the recent population, and the accuracy of pedigree- and genome-based measures was evaluated for the target population. The progenies (*n* = 1000) from each generation of the target population were extracted, and their pedigree and SNP genotyping data were used for the evaluation. In each replicate, the diagonal element of an additive relationship matrix^30^ (**A**) minus 1 was defined as the pedigree-based inbreeding coefficient, and the off-diagonal elements of **A** were defined as the pedigree-based additive relationship coefficient. Pedigree-based inbreeding coefficients and additive relationship coefficients were calculated using pedigree information traced back to the full generations (named as $\:{\mathrm{F}}_{\mathrm{A}\mathrm{P}\mathrm{E}\mathrm{D}}$ and $\:{\mathrm{f}}_{\mathrm{A}\mathrm{P}\mathrm{E}\mathrm{D}}$, respectively) and four generations (named as $\:{\mathrm{F}}_{\mathrm{P}\mathrm{E}\mathrm{D}}$ and $\:{\mathrm{f}}_{\mathrm{P}\mathrm{E}\mathrm{D}}$, respectively) in the recent population. These coefficients were calculated using the tabular method^31^ with our own program coded in R software. For genome-based measures, assuming middle-density SNP arrays, 50,000 SNPs were randomly extracted from the initial set of 60,000 founder SNPs and defined as observed SNPs. The remaining 10,000 SNPs were defined as the unobserved SNPs, because our preliminary study demonstrated that the correlation coefficients between the IBS relationships with 10,000 SNPs and 50,000 SNPs exceeded 0.90 for both inbreeding coefficients and additive relationship coefficients (Supplementary Fig. S2). The unobserved SNPs were used to calculate the reference values of the inbreeding coefficients and additive relationship coefficients. The observed SNPs were used to calculate the predicted values of the inbreeding and additive relationship coefficients. The means and SD of 100 replicates of the correlation coefficients between the reference and predicted values were then calculated.

To evaluate changes in genetic diversity over generations within the simulated population, the expected heterozygosity (*E*[Het]) was calculated using the unobserved SNPs as follows:

$$\:E\left[\mathrm{H}\mathrm{e}\mathrm{t}\right]=\frac{1}{\mathrm{m}}\sum\:_{\mathrm{j}=1}^{\mathrm{m}}2{\mathrm{p}}_{\mathrm{j}}\left(1-{\mathrm{p}}_{\mathrm{j}}\right),$$

where *m* is the number of SNPs and $\:{\mathrm{p}_\mathrm{j}}$ is the frequency of the second allele of *j*-th SNP in the target population. Unobserved SNPs are resampled per replicate, and *E*[Het] was calculated in each replicate with different combinations of *N*_*e*_ and selection criteria.

## Real cattle population

We conducted a simulation analysis based on a real cattle SNP dataset to verify our results in simulated populations. A real SNP genotype dataset was used to determine the actual extent of LD in Japanese Black cattle. Details of the experimental population and SNP information have been previously reported by Uemoto et al.^32,33^. Overall, 1368 Japanese Black cattle with SNP genotypes on the Illumina BovineHD BeadsChip (Illumina Inc., San Diego, CA, USA) were used, and the animals had no pedigree information. The dataset excluded animals that were very close relatives by excluding animals with large off-diagonal elements in the genomic relationship matrix (GRM) (cut-off value of 0.4) in the GCTA software^34^. All SNP positions were updated according to the SNPchiMp v.3 database^35^ and the ARS-UCD 1.2 reference sequence assembly was downloaded from Ensembl (release 97) (http://ftp.ensembl.org/pub/release-97/variation/vcf/bos_taurus/). The raw genotypes were phased, and the missing genotypes were imputed using Beagle5 software^36^. SNP quality control was assessed using PLINK 1.9 software^37^ with the exclusion criteria of the Hardy-Weinberg equilibrium (HWE) test with a P-value < 0.0001. After quality control, 718,223 SNPs on autosomal chromosomes were available, and these genotyped animals were regarded as the target population of the real cattle population.

In this study, 43,421 SNPs located on the Illumina BovineSNP50v2 BeadChip (Illumina Inc., San Diego, CA, USA) were extracted from all SNPs and defined as observed SNPs. The observed SNPs were used to calculate predicted values. Then, a total of 10,000 SNPs with MAF ≥ 0.01 were randomly extracted from the remaining SNPs and were defined as the unobserved SNPs. A total of 100 replicates of the extraction of unobserved SNPs were simulated, and the unobserved SNPs were used to calculate the reference values. The means and SD of the 100 replicates were calculated for the correlation coefficients between the reference and predicted values.

## Identity by state at unobserved loci

In this study, the reference values of inbreeding coefficients and additive relationship coefficients were defined as the probabilities that alleles at unobserved loci were IBS in an individual and between two individuals, respectively. Reference values were calculated as average similarity scores based on coancestry coefficients^38–40^ using 10,000 unobserved SNPs. The reference values were derived from the elements of the GRM ($\:{\mathbf{G}}_{\mathrm{t}}$), which were calculated as follows:

$$\:{\mathbf{G}}_{\mathrm{t}}=2\mathbf{K},$$

where **K** is a coancestry coefficient matrix and elements of **K** are obtained using the following steps: First, the similarity score between two individuals *x* and *y* at the *j*-th SNP ($\:{\mathrm{k}_{\mathrm{xy},\:\mathrm{j}}}$) was calculated as follows:

$$\:{\mathrm{k}}_{\mathrm{x}\mathrm{y},\:\mathrm{j}}=\frac{1}{4}\left({\mathrm{I}}_{11}+{\mathrm{I}}_{12}+{\mathrm{I}}_{21}+{\mathrm{I}}_{22}\right),$$

where $\:{\mathrm{I}_\mathrm{ab}}$ is an indicator variable equal to 1 if allele *a* on the *j*-th SNP in individual *x* and allele *b* on the same SNP in individual *y* are identical; otherwise, it is 0. Second, the average similarity score between two individuals *x* and *y* over *m* SNPs ($\:{\mathrm{k}_\mathrm{xy}}$) was calculated for all individual pairs as follows:

$$\:{\mathrm{k}}_{\mathrm{x}\mathrm{y}}=\frac{\sum\:_{\mathrm{j}=1}^{\mathrm{m}}{\mathrm{k}}_{\mathrm{x}\mathrm{y},\:\mathrm{j}}}{\mathrm{m}}.$$

The diagonal element of $\:{\mathbf{G}}_{\mathrm{t}}$ minus 1 was defined as the reference value of the inbreeding coefficient ($\:{\mathrm{F}}_{\mathrm{I}\mathrm{B}\mathrm{S}}$), and the off-diagonal elements of $\:{\mathbf{G}}_{\mathrm{t}}$ were defined as the reference values of the additive relationship coefficient ($\:{\mathrm{f}}_{\mathrm{I}\mathrm{B}\mathrm{S}}$). The $\:{\mathrm{F}}_{\mathrm{I}\mathrm{B}\mathrm{S}}$ and $\:{\mathrm{f}}_{\mathrm{I}\mathrm{B}\mathrm{S}}$ were calculated for the target populations of the simulated and real cattle populations in each replicate.

## Genome-based inbreeding coefficients and additive relationship coefficients

Using the observed SNPs, the genome-based inbreeding coefficients and additive relationship coefficients were calculated for the target populations of the simulated and real cattle populations in each replicate. We defined ten different genome-based inbreeding coefficients derived from SNP-by-SNP ($\:{\mathrm{F}}_{\mathrm{G}\mathrm{R}\mathrm{M}\mathrm{V}1}$, $\:{\mathrm{F}}_{\mathrm{G}\mathrm{R}\mathrm{M}\mathrm{V}2}$, $\:{\mathrm{F}}_{\mathrm{G}\mathrm{R}\mathrm{M}\mathrm{Y}}$, and $\:{\mathrm{F}}_{\mathrm{H}\mathrm{O}\mathrm{M}}$), haplotype ($\:{\mathrm{F}}_{\mathrm{G}\mathrm{H}\mathrm{A}\mathrm{P}}$), and homozygous segments ($\:{\mathrm{F}}_{\mathrm{R}\mathrm{O}\mathrm{H}4}$, $\:{\mathrm{F}}_{\mathrm{R}\mathrm{O}\mathrm{H}16}$, $\:{\mathrm{F}}_{\mathrm{R}\mathrm{O}\mathrm{H}4\mathrm{a}\mathrm{l}\mathrm{l}}$, $\:{\mathrm{F}}_{\mathrm{R}\mathrm{O}\mathrm{H}16\mathrm{a}\mathrm{l}\mathrm{l}}$, and $\:{\mathrm{F}}_{\mathrm{H}\mathrm{B}\mathrm{D}}$). In addition, we defined seven genome-based additive relationship coefficients derived from SNP-by-SNP ($\:{\mathrm{f}}_{\mathrm{G}\mathrm{R}\mathrm{M}\mathrm{V}1}$, $\:{\mathrm{f}}_{\mathrm{G}\mathrm{R}\mathrm{M}\mathrm{V}2}$, and $\:{\mathrm{f}}_{\mathrm{G}\mathrm{R}\mathrm{M}\mathrm{Y}}$), haplotype ($\:{\mathrm{f}}_{\mathrm{G}\mathrm{H}\mathrm{A}\mathrm{P}}$), and homozygous segments ($\:{\mathrm{f}}_{\mathrm{G}\mathrm{R}\mathrm{O}\mathrm{H}}$, $\:{\mathrm{f}}_{\mathrm{S}\mathrm{E}\mathrm{G}4}$, and $\:{\mathrm{f}}_{\mathrm{S}\mathrm{E}\mathrm{G}16}$). The details of each genome-based inbreeding coefficient and additive relationship coefficient used are described in subsequent sections below. For the simulated and real cattle populations, the observed SNPs were assessed using the exclusion criterion of MAF < 0.01 in the target population with the except for $\:{\mathrm{F}}_{\mathrm{R}\mathrm{O}\mathrm{H}4\mathrm{a}\mathrm{l}\mathrm{l}}$ and $\:{\mathrm{F}}_{\mathrm{R}\mathrm{O}\mathrm{H}16\mathrm{a}\mathrm{l}\mathrm{l}}$. The number of SNPs with MAF ≥ 0.01 was 34,503 in the real cattle populations. Using the observed SNPs, genome-based measures were calculated as follows:

$\:{\mathrm{F}}_{\mathrm{G}\mathrm{R}\mathrm{M}\mathrm{V}1}$, $\:{\mathrm{F}}_{\mathrm{G}\mathrm{R}\mathrm{M}\mathrm{V}2}$, $\:{\mathrm{f}}_{\mathrm{G}\mathrm{R}\mathrm{M}\mathrm{V}1}$, and $\:{\mathrm{f}}_{\mathrm{G}\mathrm{R}\mathrm{M}\mathrm{V}2}$ were derived from VanRaden’s GRM^41^ ($\:{\mathbf{G}}_{\mathrm{V}}$), which was calculated as follows:

1 $$\:{\mathbf{G}}_{\mathrm{V}}=\frac{\mathbf{Z}{\mathbf{Z}}^{{\prime\:}}}{2\sum\:_{\mathrm{j}=1}^{\mathrm{m}}{\mathrm{p}}_{\mathrm{j}}\left(1-{\mathrm{p}}_{\mathrm{j}}\right)},$$

where *m* is the number of SNPs, $\:{\mathrm{p}_\mathrm{j}}$ is the frequency of the second allele of *j*-th SNP, and the elements of **Z** are calculated as $\:{\mathrm{z}}_{\mathrm{i}\mathrm{j}}={\mathrm{x}}_{\mathrm{i}\mathrm{j}}-2{\mathrm{p}}_{\mathrm{j}}$, where $\:{\mathrm{x}}_{\mathrm{i}\mathrm{j}}$ is the number of second alleles of the *i*-th individual at the *j*-th SNP. When $\:{\mathrm{p}_\mathrm{j}}$ is calculated in the target population, the diagonal elements of $\:{\mathbf{G}}_{\mathrm{V}}$ minus 1 are defined as $\:{\mathrm{F}}_{\mathrm{G}\mathrm{R}\mathrm{M}\mathrm{V}1}$ and the off-diagonal elements of $\:{\mathbf{G}}_{\mathrm{V}}$ are defined as $\:{\mathrm{f}}_{\mathrm{G}\mathrm{R}\mathrm{M}\mathrm{V}1}$. When $\:{\mathrm{p}_\mathrm{j}}$ is set to 0.5, which is assumed to be the frequency of the founders, the diagonal element of $\:{\mathbf{G}}_{\mathrm{V}}$ minus one is defined as $\:{\mathrm{F}}_{\mathrm{G}\mathrm{R}\mathrm{M}\mathrm{V}2}$ and the off-diagonal elements of $\:{\mathbf{G}}_{\mathrm{V}}$ are defined as $\:{\mathrm{f}}_{\mathrm{G}\mathrm{R}\mathrm{M}\mathrm{V}2}$. These coefficients were based on the assumption that rare homozygous genotypes contribute more to the inbreeding measure than common homozygous genotypes^42^.

$\:{\mathrm{F}}_{\mathrm{G}\mathrm{R}\mathrm{M}\mathrm{Y}}$ and $\:{\mathrm{f}}_{\mathrm{G}\mathrm{R}\mathrm{M}\mathrm{Y}}$ were derived from Yang’s GRM^43^ ($\:{\mathbf{G}}_{\mathrm{Y}}$), which was calculated as follows:

$$\:{\mathbf{G}}_{\mathrm{Y}}=\frac{\mathbf{W}{\mathbf{W}}^{{\prime\:}}}{\mathrm{m}},$$

where the elements of **W** are calculated as $\:{\mathrm{w}}_{\mathrm{i}\mathrm{j}}=\left({\mathrm{x}}_{\mathrm{i}\mathrm{j}}-2{\mathrm{p}}_{\mathrm{j}}\right)/\sqrt{2{\mathrm{p}}_{\mathrm{j}}\left(1-{\mathrm{p}}_{\mathrm{j}}\right)}$. When $\:{\mathrm{p}_\mathrm{j}}$ was calculated in the target population, the diagonal elements of $\:{\mathbf{G}}_{\mathrm{Y}}$ minus 1 were defined as $\:{\mathrm{F}}_{\mathrm{G}\mathrm{R}\mathrm{M}\mathrm{Y}}$ and the off-diagonal elements of $\:{\mathbf{G}}_{\mathrm{Y}}$ were defined as $\:{\mathrm{f}}_{\mathrm{G}\mathrm{R}\mathrm{M}\mathrm{Y}}$. These coefficients are based on the correlation of uniting gametes^43,44^ and also give more weight to homozygotes for the minor allele than to homozygotes for the major allele^45^.

$\:{\mathrm{F}}_{\mathrm{H}\mathrm{O}\mathrm{M}}$ is based on excess homozygosity^46^ and is calculated as follows:

$$\:{\mathrm{F}}_{\mathrm{H}\mathrm{O}\mathrm{M}}=\frac{O\left[\mathrm{h}\mathrm{o}\mathrm{m}\right]-E\left[\mathrm{h}\mathrm{o}\mathrm{m}\right]}{\mathrm{m}-E\left[\mathrm{h}\mathrm{o}\mathrm{m}\right]},$$

where *O*[hom] and *E*[hom] are the observed and expected numbers of homozygous SNPs in an individual under HWE, respectively. $\:{\mathrm{F}}_{\mathrm{H}\mathrm{O}\mathrm{M}}$ was calculated using the PLINK software^37^, and *E*[hom] was calculated from the allele frequency of the target population.

$\:{\mathrm{F}}_{\mathrm{G}\mathrm{H}\mathrm{A}\mathrm{P}}$ and $\:{\mathrm{f}}_{\mathrm{G}\mathrm{H}\mathrm{A}\mathrm{P}}$ were based on haplotype information. A haplotype is defined as a group of closely linked SNPs on the same homologous chromosomes that are frequently inherited. These measures were calculated using the following steps: First, the phased SNP genotypes for all individuals were obtained from the outputs of QMSim software^24^ in the simulated cattle population and those of Beagle5 software^36^ in the real cattle population and were then used for the analysis. The LD coefficient (r^2^) values^29^ were calculated to evaluate the LD between SNPs and to construct haploblocks with a threshold of 0.25 (the default value) based on the big-LD approach using the *GPART* package in R software^47^. In this study, a haploblock was defined as a genomic region spanning at least two SNPs. Second, the haplotype alleles were converted to pseudo-SNPs^48^, and each pseudo-SNP allele corresponded to one of the unique haplotype alleles present within a haploblock. The number of copies of a specific pseudo-SNP allele was counted and coded as 0, 1, or 2 for each individual, similar to the code used for SNP. Pseudo-SNPs were assessed using the exclusion criterion of MAF < 0.01. Third, pseudo-SNPs were used to construct a haplotype-based GRM ($\:{\mathbf{G}}_{\mathrm{H}}$) based on Eq. (1), with $\:{\mathrm{p}_\mathrm{j}}$ calculated for the target population. The diagonal element of $\:{\mathbf{G}}_{\mathrm{H}}$ minus one is defined as $\:{\mathrm{F}}_{\mathrm{G}\mathrm{H}\mathrm{A}\mathrm{P}}$ and the off-diagonal element of $\:{\mathbf{G}}_{\mathrm{H}}$ is defined as $\:{\mathrm{f}}_{\mathrm{G}\mathrm{H}\mathrm{A}\mathrm{P}}$.

$\:{\mathrm{F}}_{\mathrm{R}\mathrm{O}\mathrm{H}4}$, $\:{\mathrm{F}}_{\mathrm{R}\mathrm{O}\mathrm{H}4\mathrm{a}\mathrm{l}\mathrm{l}}$, $\:{\mathrm{F}}_{\mathrm{R}\mathrm{O}\mathrm{H}16}$, and $\:{\mathrm{F}}_{\mathrm{R}\mathrm{O}\mathrm{H}16\mathrm{a}\mathrm{l}\mathrm{l}}$ are based on run of homozygosity (ROH). ROH are defined as contiguous homozygous stretches in an individual genome, resulting from the transmission of identical haplotypes from parents to offspring^49,50^. The identification and characterization of ROH in a population can provide insights into how the population structure has evolved over time. In this study, ROH was estimated using PLINK software^37^, which uses a sliding window approach to define ROH as a stretch that includes a minimum specified number of homozygous SNPs within a specified distance. We applied the following parameters and thresholds to define an ROH: (i) a sliding window size of 50 SNPs, (ii) the number of heterozygous SNPs allowed in the ROH was 1, (iii) the maximum allowed distance between consecutive SNPs was 1 Mbp, (iv) the minimum density of SNP in a sliding window was 1 SNP every 100 kbp, (v) the minimum length of an ROH was set to 4 Mbp and 16 Mbp, and (vi) the minimum number of consecutive homozygous SNP included in the ROH (*L*), which was calculated in each target population as follows:

$$\:\mathrm{L}=\frac{ln\frac{{\upalpha\:}}{{\mathrm{n}}_{\mathrm{s}}{\mathrm{n}}_{\mathrm{i}}}}{ln\left(1-\stackrel{-}{\mathrm{h}\mathrm{e}\mathrm{t}}\right)},$$

where α is the false positive probability set to 0.05, n_s_ is the number of SNPs per individual, n_i_ is the number of SNP genotyped individual, and $\:\stackrel{-}{\mathrm{h}\mathrm{e}\mathrm{t}}$ is the mean of heterozygosity across all SNPs^51,52^. The inbreeding coefficient based on ROH was defined as the total length of the ROH divided by the overall length of the autosomal genome^50^. The overall length of the autosomal genome was defined as 2,489,385 kbp based on the ARS-UCD 1.2 reference sequence assembly (https://www.ncbi.nlm.nih.gov/datasets/genome/GCF_002263795.1/). The scale of the inter-marker distance (cM) in the simulated cattle population was assumed to be in Mbp. In this study, four different types of ROH-based inbreeding coefficients ($\:{\mathrm{F}}_{\mathrm{R}\mathrm{O}\mathrm{H}4}$, $\:{\mathrm{F}}_{\mathrm{R}\mathrm{O}\mathrm{H}4\mathrm{a}\mathrm{l}\mathrm{l}}$, $\:{\mathrm{F}}_{\mathrm{R}\mathrm{O}\mathrm{H}16}$and $\:{\mathrm{F}}_{\mathrm{R}\mathrm{O}\mathrm{H}16\mathrm{a}\mathrm{l}\mathrm{l}}$) were defined based on the size of the ROH and MAF conditions. The size of the ROH is inversely correlated with its age: longer ROH originate from recent common ancestors while shorter ROH come from ancient ancestors because they have been broken down by recombination over many generations^53^. Two thresholds for the size of the ROH from ancient and recent ancestors were defined based on the minimum length of an ROH (4 Mbp or 16 Mbp, respectively). The $\:{\mathrm{F}}_{\mathrm{R}\mathrm{O}\mathrm{H}4}$ and $\:{\mathrm{F}}_{\mathrm{R}\mathrm{O}\mathrm{H}4\mathrm{a}\mathrm{l}\mathrm{l}}$ used SNPs with MAF ≥ 0.01 and all SNPs, respectively, to estimate the ROH with a minimum length of 4 Mbp. The $\:{\mathrm{F}}_{\mathrm{R}\mathrm{O}\mathrm{H}16}$ and $\:{\mathrm{F}}_{\mathrm{R}\mathrm{O}\mathrm{H}16\mathrm{a}\mathrm{l}\mathrm{l}}$ used SNPs with MAF ≥ 0.01 and all SNPs, respectively, to estimate the ROH with a minimum length of 16 Mbp.

$\:{\mathrm{f}}_{\mathrm{G}\mathrm{R}\mathrm{O}\mathrm{H}}$ was derived from the ROH-based GRM^54^ ($\:{\mathbf{G}}_{\mathrm{R}\mathrm{O}\mathrm{H}}$), and indicates whether animals are inbred at the same genomic positions^54^. To quantify the effect of an SNP in an ROH, the SNP genotype in an ROH and not being an ROH was set to 1 and 0, respectively, for each individual. Then, $\:{\mathbf{G}}_{\mathrm{R}\mathrm{O}\mathrm{H}}$ was calculated as follows:

$$\:{\mathbf{G}}_{\mathrm{R}\mathrm{O}\mathrm{H}}=\frac{\mathbf{Y}{\mathbf{Y}}^{{\prime\:}}}{\sum\:_{\mathrm{j}=1}^{\mathrm{m}}{\mathrm{q}}_{\mathrm{j}}\left(1-{\mathrm{q}}_{\mathrm{j}}\right)},$$

where the elements of **Y** ($\:{\mathrm{y}}_{\mathrm{i}\mathrm{j}}$) are $\:1-{\mathrm{q}}_{\mathrm{j}}$ for being in an ROH or $\:0-{\mathrm{q}}_{\mathrm{j}}$ for not being in an ROH of the *i*-th individual at the *j*-th SNP, and $\:{\mathrm{q}_\mathrm{j}}$ is the frequency of *j*-th SNP being in a ROH. In this study, ROH was estimated using PLINK software^37^ with the same parameters and thresholds as $\:{\mathrm{F}}_{\mathrm{R}\mathrm{O}\mathrm{H}4}$ to define ROH. After calculating $\:{\mathbf{G}}_{\mathrm{R}\mathrm{O}\mathrm{H}}$, the off-diagonal elements of $\:{\mathbf{G}}_{\mathrm{R}\mathrm{O}\mathrm{H}}$ were defined as $\:{\mathrm{f}}_{\mathrm{G}\mathrm{R}\mathrm{O}\mathrm{H}}$.

$\:{\mathrm{F}}_{\mathrm{H}\mathrm{B}\mathrm{D}}$ is based on homozygous-by-descent^55^ (HBD). HBD segments are chromosomal segments inherited twice from a common ancestor without recombination. HBD segments result in long stretches of homozygous genotypes (i.e., ROH), and the ROH segment is autozygous. The classification of HBD segments was estimated using the *RZooRoH* package in R software^56^, which is a model-based approach based on a Hidden Markov Model (HMM) to identify HBD segments^57^. In this model, the genomic region is described as a mosaic of HBD and non-HBD segments with HMM. The age of inbreeding is estimated for HBD classes based on the transition probability between different HBD and non-HBD segments and is conditional on class specificity. The probability of staying in a particular state is calculated as $\:{\mathrm{e}}^{-{R}_{k}}$, where *R*_*k*_ is the rate specific to the *k*-th class. This implies that the length of an HBD segment of any class follows an exponential distribution with rate *R*_*k*_. In this study, 10 HBD classes (the default settings) were used, and the proportion of HBD loci to all loci was calculated and defined as $\:{\mathrm{F}}_{\mathrm{H}\mathrm{B}\mathrm{D}}$.

$\:{\mathrm{f}}_{\mathrm{S}\mathrm{E}\mathrm{G}4}$ and $\:{\mathrm{f}}_{\mathrm{S}\mathrm{E}\mathrm{G}16}$ are additive relationship coefficients based on shared homozygous segments between two individuals ($\:{\mathrm{f}}_{\mathrm{S}\mathrm{E}\mathrm{G}}$), which are interpreted as the proportion of ROH sharing between two individuals^58,59^. The $\:{\mathrm{f}}_{\mathrm{S}\mathrm{E}\mathrm{G}}$ for individuals *x* and *y* ($\:{\mathrm{f}}_{\mathrm{S}\mathrm{E}\mathrm{G}}\left(\mathrm{x},\:\mathrm{y}\right)$) is defined as follows:

$$\:{\mathrm{f}}_{\mathrm{S}\mathrm{E}\mathrm{G}}\left(\mathrm{x},\:\mathrm{y}\right)=\frac{\sum\:_{\mathrm{k}}{\sum\:}_{{\mathrm{a}}_{\mathrm{x}}=1}^{2}{\sum\:}_{{\mathrm{b}}_{\mathrm{y}}=1}^{2}\left\{{\mathrm{L}}_{{\mathrm{s}\mathrm{e}\mathrm{g}}_{\mathrm{k}}}\left({\mathrm{a}}_{\mathrm{x}},\:{\mathrm{b}}_{\mathrm{y}}\right)\right\}}{4\mathrm{L}},$$

where *L* is the total length of the genome, $\:{\mathrm{L}_{\mathrm{seg}_\mathrm{k}}\left(\mathrm{a}_\mathrm{x},\:\mathrm{b}_\mathrm{y}\right)}$ is the *k*-th ROH region between chromosome *a* of individual *x* and chromosome *b* of individual *y*. The $\:{\mathrm{f}}_{\mathrm{S}\mathrm{E}\mathrm{G}}$ was predicted using the *optisel* package in R software^59^. We calculated $\:{\mathrm{f}}_{\mathrm{S}\mathrm{E}\mathrm{G}4}$ and $\:{\mathrm{f}}_{\mathrm{S}\mathrm{E}\mathrm{G}16}$ for all pairs of individuals in the target population using the following parameters: the minimum number of SNP in $\:{\mathrm{L}_{\mathrm{seg}_\mathrm{k}}\left(\mathrm{a}_\mathrm{x},\:\mathrm{b}_\mathrm{y}\right)}$ was 50, and the minimum length of $\:{\mathrm{L}_{\mathrm{seg}_\mathrm{k}}\left(\mathrm{a}_\mathrm{x},\:\mathrm{b}_\mathrm{y}\right)}$ was 4 and 16 Mbp, respectively.

## Results

### Simulated cattle population

**Fig. 2:**
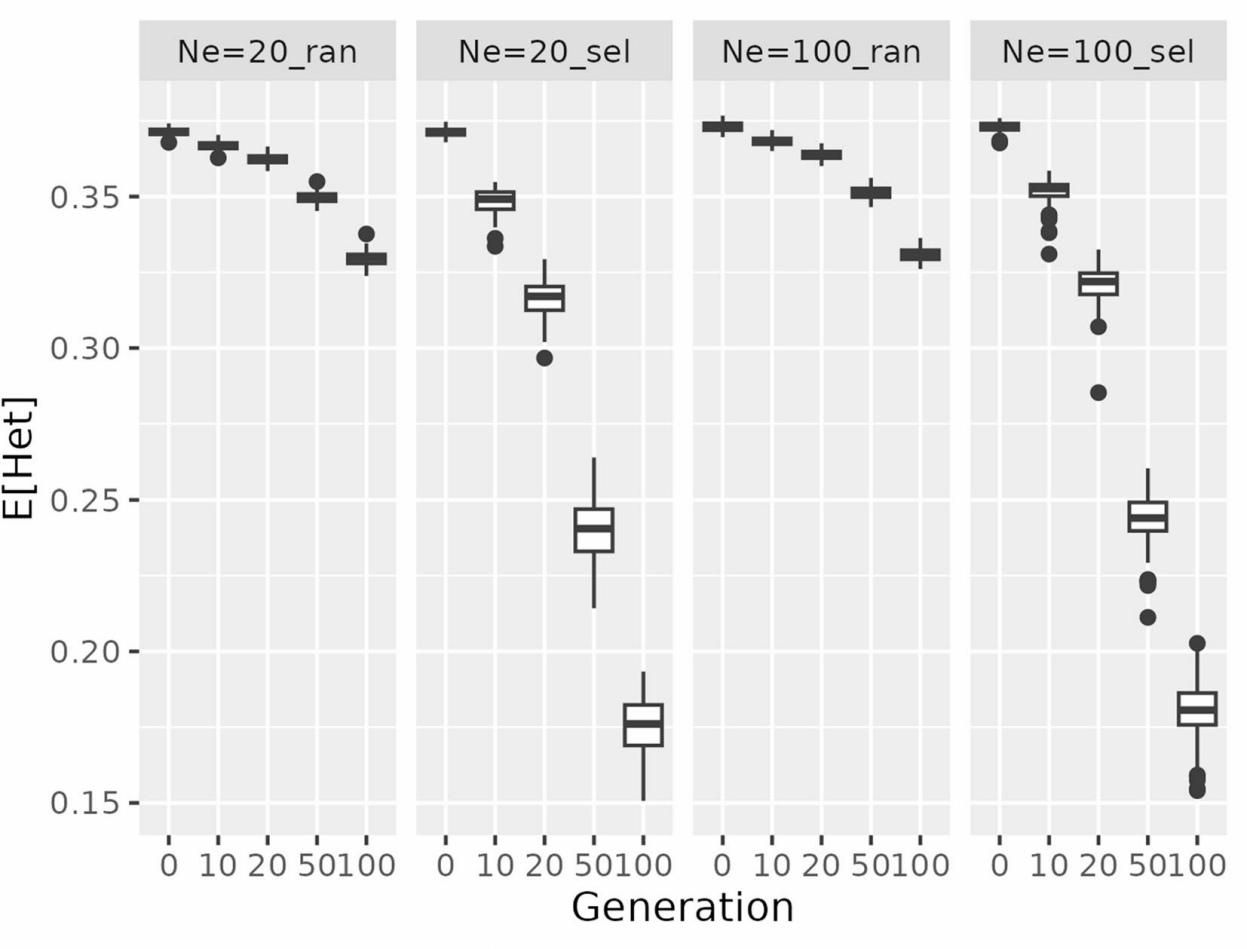
Trends of expected heterozygosity in the simulated cattle population. Expected heterozygosity (*E*[Het]) derived from 10,000 unobserved single nucleotide polymorphisms (SNPs) was calculated at generations 10, 20, 50, and 100 in a simulated cattle population with four combinations of effective population size (*N*_*e*_=20 and *N*_*e*_=100) and selection criteria (ran: selection at random and sel: selection based on estimated breeding values) in each replicate.

To evaluate the effect of *N*_*e*_ and selection criteria on the genomic structure of the simulated cattle population, Fig. 2 shows the trends in expected heterozygosity derived from 10,000 unobserved SNPs for populations with four different combinations of *N*_*e*_ (*N*_*e*_ =20 or *N*_*e*_ =100) and selection criteria (selection at random or selection based on EBVs). The results of this study indicated that expected heterozygosity declined over generations across all simulated populations. The magnitude of this decline was more pronounced in populations subjected to selection based on EBVs than in those subjected to random selection. Additionally, the difference of the expected heterozygosity with *N*_*e*_ =20 and *N*_*e*_ =100 was modest in later generations under selection. The mean ± SD of *E*[Het] in G100 were 0.175 ± 0.009 and 0.180 ± 0.010 in *N*_*e*_ =20_sel and *N*_*e*_ =100_sel, respectively. It is generally accepted that a smaller *N*_*e*_ should accelerate genetic drift and reduce heterozygosity more rapidly. This result may be indicative of the simulation settings or a limited sensitivity of the heterozygosity metric used. In the simulation settings, the progeny size per generation is moderate (*n* = 1,000) in the recent population, and the selection intensity is low (from 500 to 50 in male and no selection intensity in female). Selection is also based on overlapping generations with low replacement rate (0.5 for males and 0.2 for females). Our setting values of overlapping generations and selection/replacement conditions masked differences in *N*_*e*_ within the time frame, which may have contributed to the modest differences in the expected heterozygosity observed in our results.

**Fig. 3:**
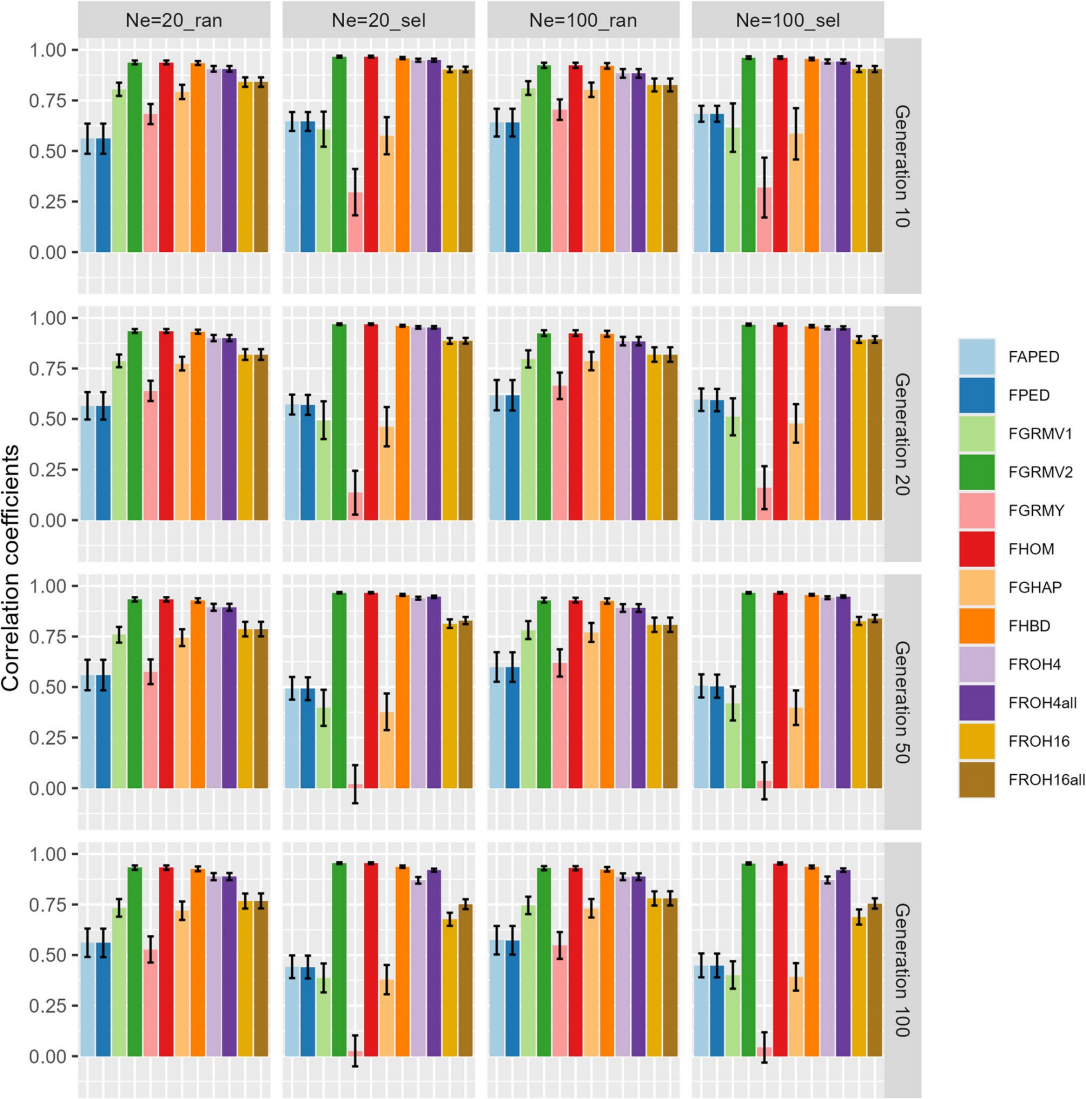
Correlation coefficients between reference and predicted values of inbreeding coefficients in the simulated cattle population. Correlation coefficients were calculated at generations 10, 20, 50, and 100 in the simulated cattle population with four combinations of effective population size (Ne = 20 and Ne = 100) and selection criteria (ran: selection at random and sel: selection based on estimated breeding values) in each replicate. Mean and SD of 100 replicates were calculated and plotted. The calculations of the reference and predicted values of inbreeding coefficients are explained in the main text. The abbreviations of the pedigree- and genome-based measures are also explained in the main text.

The correlation coefficients between the reference and predicted values of the inbreeding coefficients in the target population of the simulated cattle are presented in Fig. 3 and Supplementary Table S2. There were no notable differences in the correlation coefficients between the *N*_*e*_ =20_ran and *N*_*e*_ =100_ran scenarios and between the *N*_*e*_ =20_sel and *N*_*e*_ =100_sel scenarios. In the population subjected to selection at random, the correlation coefficients ranged from 0.56 to 0.64 in pedigree-based measures and from 0.53 to 0.94 in genome-based measures. In particular, $\:{\mathrm{F}}_{\mathrm{G}\mathrm{R}\mathrm{M}\mathrm{V}2}$, $\:{\mathrm{F}}_{\mathrm{H}\mathrm{O}\mathrm{M}}$, and $\:{\mathrm{F}}_{\mathrm{H}\mathrm{B}\mathrm{D}}$ exhibited correlation coefficients exceeding 0.90 in all generations. There were no large differences in the correlation coefficients between generations for any of the measures. In the population subjected to selection based on EBVs, the correlation coefficients were ranged from 0.44 to 0.68 in pedigree-based measures and from 0.02 to 0.97 in genome-based measures. Notably, while the correlation coefficients for many of the measures declined over the generations, those for $\:{\mathrm{F}}_{\mathrm{G}\mathrm{R}\mathrm{M}\mathrm{V}2}$, $\:{\mathrm{F}}_{\mathrm{H}\mathrm{O}\mathrm{M}}$, $\:{\mathrm{F}}_{\mathrm{H}\mathrm{B}\mathrm{D}}$, and $\:{\mathrm{F}}_{\mathrm{R}\mathrm{O}\mathrm{H}4\mathrm{a}\mathrm{l}\mathrm{l}}$ remained constant, exceeding 0.90 for all generations. In contrast, the correlation coefficients for $\:{\mathrm{F}}_{\mathrm{G}\mathrm{R}\mathrm{M}\mathrm{Y}}$ were the lowest, ranging from 0.02 to 0.32. The correlation coefficients for $\:{\mathrm{F}}_{\mathrm{R}\mathrm{O}\mathrm{H}4}$, $\:{\mathrm{F}}_{\mathrm{R}\mathrm{O}\mathrm{H}4\mathrm{a}\mathrm{l}\mathrm{l}}$, $\:{\mathrm{F}}_{\mathrm{R}\mathrm{O}\mathrm{H}16}$ and $\:{\mathrm{F}}_{\mathrm{R}\mathrm{O}\mathrm{H}16\mathrm{a}\mathrm{l}\mathrm{l}}$, the correlation coefficients for $\:{\mathrm{F}}_{\mathrm{R}\mathrm{O}\mathrm{H}4}$ and $\:{\mathrm{F}}_{\mathrm{R}\mathrm{O}\mathrm{H}4\mathrm{a}\mathrm{l}\mathrm{l}}$ were higher than those for $\:{\mathrm{F}}_{\mathrm{R}\mathrm{O}\mathrm{H}16}$ and $\:{\mathrm{F}}_{\mathrm{R}\mathrm{O}\mathrm{H}16\mathrm{a}\mathrm{l}\mathrm{l}}$, respectively, in all scenarios. Moreover, the correlation coefficients for $\:{\mathrm{F}}_{\mathrm{R}\mathrm{O}\mathrm{H}4\mathrm{a}\mathrm{l}\mathrm{l}}$ and $\:{\mathrm{F}}_{\mathrm{R}\mathrm{O}\mathrm{H}16\mathrm{a}\mathrm{l}\mathrm{l}}$ were higher than those for $\:{\mathrm{F}}_{\mathrm{R}\mathrm{O}\mathrm{H}4}$ and $\:{\mathrm{F}}_{\mathrm{R}\mathrm{O}\mathrm{H}16}$, respectively, in the later generation in the population with selection based on EBVs, while there were no differences in the coefficients between SNPs with MAF ≥ 0.01 and all SNPs in the other populations.

Table 2 shows the mean values of inbreeding coefficients for the target simulated cattle populations. There were no notable differences in the inbreeding coefficients between the *N*_*e*_ =20 and *N*_*e*_ =100 scenarios. The $\:{\mathrm{F}}_{\mathrm{I}\mathrm{B}\mathrm{S}}$, $\:{\mathrm{F}}_{\mathrm{A}\mathrm{P}\mathrm{E}\mathrm{D}}$, $\:{\mathrm{F}}_{\mathrm{G}\mathrm{R}\mathrm{M}\mathrm{V}2}$, $\:{\mathrm{F}}_{\mathrm{H}\mathrm{B}\mathrm{D}}$, and $\:{\mathrm{F}}_{\mathrm{R}\mathrm{O}\mathrm{H}4\mathrm{a}\mathrm{l}\mathrm{l}}$, and $\:{\mathrm{F}}_{\mathrm{R}\mathrm{O}\mathrm{H}16\mathrm{a}\mathrm{l}\mathrm{l}}$ increased over generations in all scenarios, particularly in the population selected based on EBVs. In contrast, $\:{\mathrm{F}}_{\mathrm{G}\mathrm{R}\mathrm{M}\mathrm{V}1}$, $\:{\mathrm{F}}_{\mathrm{G}\mathrm{R}\mathrm{M}\mathrm{Y}}$, $\:{\mathrm{F}}_{\mathrm{H}\mathrm{O}\mathrm{M}}$, and $\:{\mathrm{F}}_{\mathrm{G}\mathrm{H}\mathrm{A}\mathrm{P}}$ were constantly around zero over generations in all scenarios, and the $\:{\mathrm{F}}_{\mathrm{P}\mathrm{E}\mathrm{D}}$, $\:{\mathrm{F}}_{\mathrm{R}\mathrm{O}\mathrm{H}4}$, and $\:{\mathrm{F}}_{\mathrm{R}\mathrm{O}\mathrm{H}16}$ decreased from the later generations in the population with selection based on EBVs.

**Table 2:** Mean values of inbreeding coefficients in the simulated cattle population. Inbreeding coefficients were calculated at generation 10 (G10), 20 (G20), 50 (G50), and 100 (G100) in the simulated cattle population with four combinations of effective population size (Ne=20 and Ne=100) and selection criteria (ran: selection at random and sel: selection based on estimated breeding values) in each replicate. Mean values of 100 replicates were calculated. The calculations of the reference and predicted values of inbreeding coefficients are explained in the main text. The abbreviations of the pedigree- and genome-based measures are also explained in the main text.

Measures	Ne=20_ran				Ne=20_sel			
	G10	G20	G50	G100	G10	G20	G50	G100
F_IBS_	0.63	0.64	0.65	0.67	0.65	0.68	0.76	0.82
F_APED_	0.01	0.02	0.06	0.11	0.05	0.11	0.24	0.37
F_PED_	0.01	0.02	0.02	0.02	0.05	0.06	0.05	0.04
F_GRMV1_	0.00	0.00	0.00	0.00	−0.01	−0.01	0.00	0.00
F_GRMV2_	0.26	0.27	0.28	0.30	0.29	0.32	0.36	0.37
F_GRMY_	0.00	0.00	0.00	0.00	−0.01	−0.01	0.00	0.00
F_HOM_	0.00	0.00	0.00	0.00	−0.01	−0.01	0.00	0.00
F_GHAP_	0.00	0.00	0.00	0.00	−0.01	−0.01	0.00	0.00
F_HBD_	0.23	0.23	0.24	0.27	0.25	0.29	0.39	0.46
F_ROH4_	0.09	0.10	0.12	0.14	0.13	0.20	0.33	0.29
F_ROH4all_	0.09	0.10	0.12	0.14	0.13	0.21	0.37	0.51
F_ROH16_	0.04	0.04	0.03	0.03	0.07	0.11	0.15	0.08
F_ROH16all_	0.04	0.04	0.03	0.03	0.07	0.11	0.19	0.24

Measures	Ne=100_ran				Ne=100_sel			
	G10	G20	G50	G100	G10	G20	G50	G100
F_IBS_	0.63	0.63	0.65	0.67	0.63	0.68	0.76	0.82
F_APED_	0.01	0.02	0.06	0.11	0.01	0.11	0.24	0.37
F_PED_	0.01	0.02	0.02	0.02	0.01	0.06	0.05	0.04
F_GRMV1_	0.00	0.00	0.00	0.00	0.00	−0.01	0.00	0.00
F_GRMV2_	0.26	0.27	0.28	0.30	0.26	0.32	0.36	0.37
F_GRMY_	0.00	0.00	0.00	0.00	0.00	−0.01	0.00	0.00
F_HOM_	0.00	0.00	0.00	0.00	0.00	−0.01	0.00	0.00
F_GHAP_	0.00	0.00	0.00	0.00	0.00	−0.01	0.00	0.00
F_HBD_	0.21	0.21	0.23	0.25	0.21	0.27	0.37	0.44
F_ROH4_	0.06	0.07	0.09	0.12	0.06	0.17	0.32	0.29
F_ROH4all_	0.06	0.07	0.09	0.12	0.06	0.17	0.35	0.49
F_ROH16_	0.02	0.03	0.03	0.03	0.02	0.10	0.15	0.09
F_ROH16all_	0.02	0.03	0.03	0.03	0.02	0.10	0.18	0.22

**Fig. 4:**
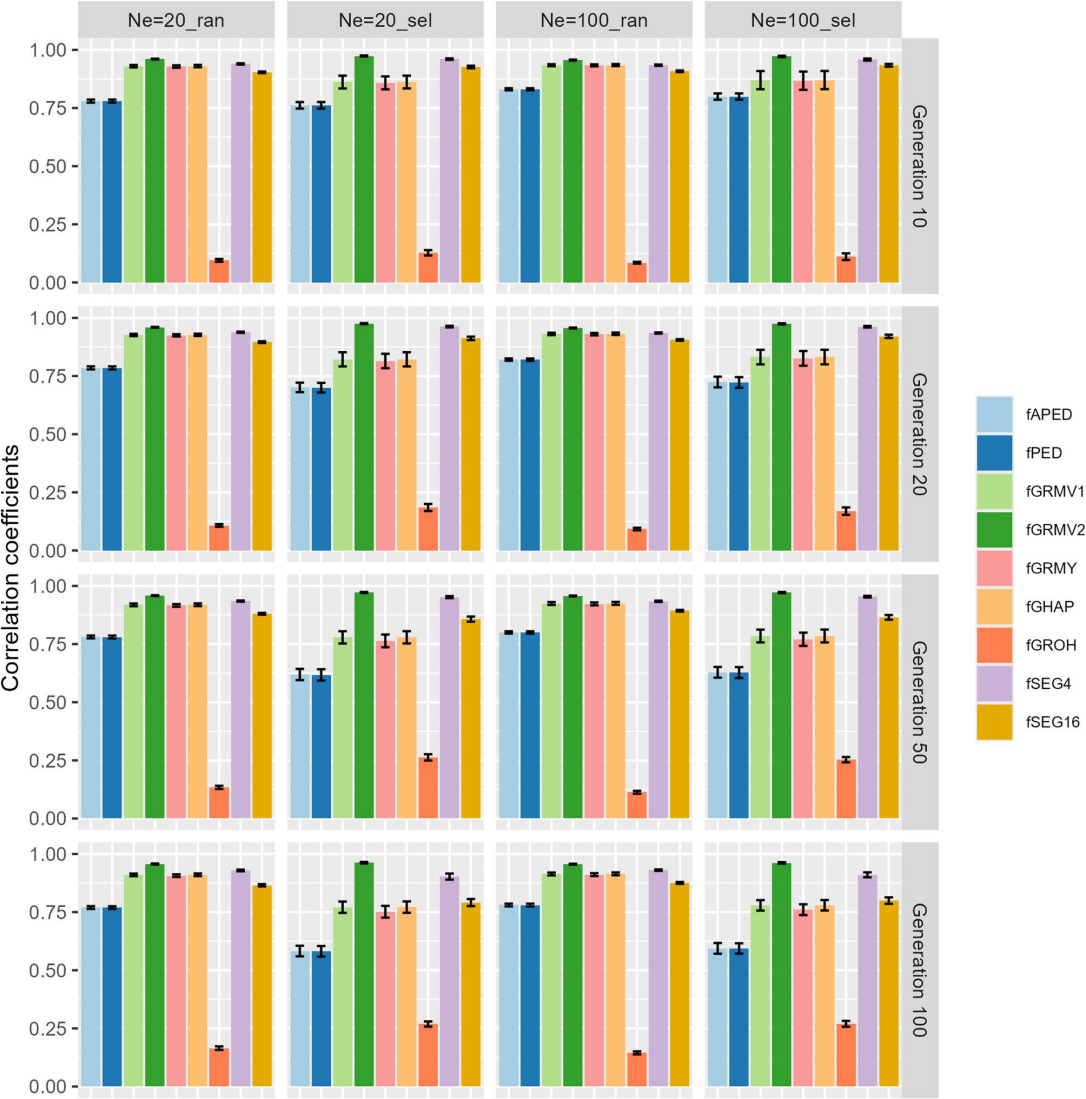
Correlation coefficients between reference and predicted values of additive relationship coefficients in the simulated cattle population. Correlation coefficients were calculated at generation 10, 20, 50, and 100 in the simulated cattle population with four combinations of effective population size (*N*_*e*_=20 and *N*_*e*_=100) and selection criteria (ran: selection at random and sel: selection based on estimated breeding values) in each replicate. Mean and SD of 100 replicates were calculated and plotted. The calculations of the reference and predicted values of additive relationship coefficients are explained in the main text. The abbreviations of the pedigree- and genome-based measures are also explained in the main text.

The correlation coefficients between the reference and predicted values of the additive relationship coefficients in the target population of simulated cattle are shown in Fig. 4 and Supplementary Table S3. There were no notable differences in the correlation coefficients between the *N*_*e*_ =20 and *N*_*e*_ =100 scenarios. In the population subjected to selection at random, the correlation coefficients ranged from 0.77 to 0.83 in pedigree-based measures and from 0.08 to 0.96 in genome-based measures. In particular, all genome-based measures, except for $\:{\mathrm{f}}_{\mathrm{G}\mathrm{R}\mathrm{O}\mathrm{H}}$ and $\:{\mathrm{f}}_{\mathrm{S}\mathrm{E}\mathrm{G}16}$ had correlation coefficients exceeding 0.90 in all generations, and those for $\:{\mathrm{f}}_{\mathrm{G}\mathrm{R}\mathrm{O}\mathrm{H}}$ exhibited the lowest values, ranging from 0.08 to 0.17. There were no large differences in the correlation coefficients between generations for any of the measures. In the population subjected to selection based on EBVs, the correlation coefficients ranged from 0.58 to 0.80 in pedigree-based measures and from 0.11 to 0.98 in genome-based measures. Notably, while the correlation coefficients for many of the measures exhibited a decline over generations, the correlation coefficients for $\:{\mathrm{f}}_{\mathrm{G}\mathrm{R}\mathrm{M}\mathrm{V}2}$ and $\:{\mathrm{f}}_{\mathrm{S}\mathrm{E}\mathrm{G}4}$ exceeded 0.90 for all generations. Conversely, the correlation coefficients for $\:{\mathrm{f}}_{\mathrm{G}\mathrm{R}\mathrm{O}\mathrm{H}}$ were the lowest, ranging from 0.11 to 0.27.

## Real cattle population

**Fig. 5:**
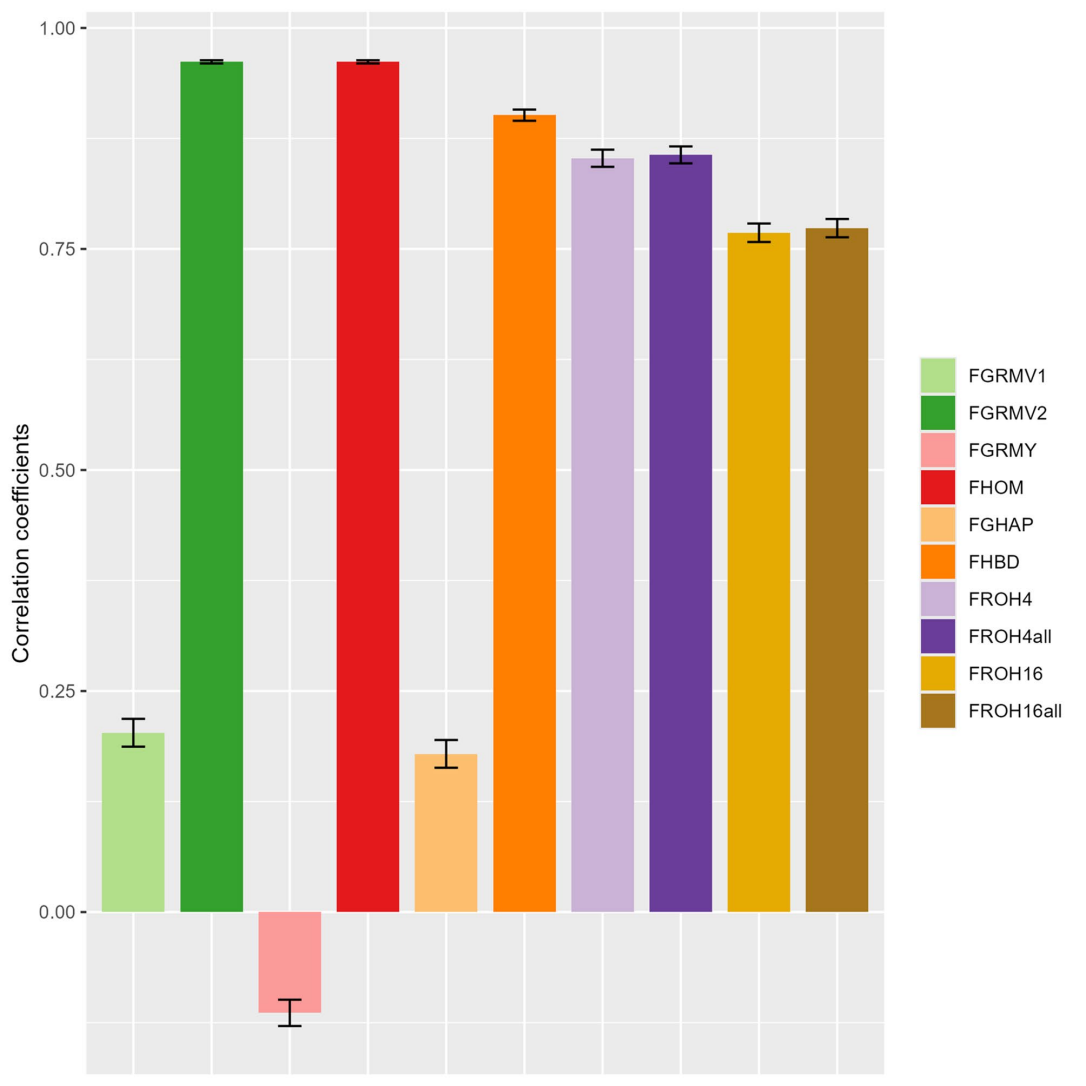
Correlation coefficients between reference and predicted values of inbreeding coefficients in the real cattle population. Correlation coefficients were calculated in the real cattle population, which was simulated by a total of 100 replicates of random extraction for the unobserved single nucleotide polymorphisms (SNPs). Mean and SD of 100 replicates were calculated and plotted. The calculations of the reference and predicted values of inbreeding coefficients are explained in the main text. The abbreviations of the genome-based measures are also explained in the main text.

The correlation coefficients between the reference and predicted values of the inbreeding coefficients in a real cattle population are shown in Fig. 5 and Supplementary Table S4. The correlation coefficients ranged from − 0.11 to 0.96, with $\:{\mathrm{F}}_{\mathrm{G}\mathrm{R}\mathrm{M}\mathrm{V}2}$ and $\:{\mathrm{F}}_{\mathrm{H}\mathrm{O}\mathrm{M}}$ exhibiting the highest coefficients (0.96). $\:{\mathrm{F}}_{\mathrm{H}\mathrm{B}\mathrm{D}}$, $\:{\mathrm{F}}_{\mathrm{R}\mathrm{O}\mathrm{H}4}$, and $\:{\mathrm{F}}_{\mathrm{R}\mathrm{O}\mathrm{H}4\mathrm{a}\mathrm{l}\mathrm{l}}$ exhibited high coefficients (ranging from 0.85 to 0.90), $\:{\mathrm{F}}_{\mathrm{R}\mathrm{O}\mathrm{H}16}$, and $\:{\mathrm{F}}_{\mathrm{R}\mathrm{O}\mathrm{H}16\mathrm{a}\mathrm{l}\mathrm{l}}$ exhibited moderate coefficients (0.77), and $\:{\mathrm{F}}_{\mathrm{G}\mathrm{R}\mathrm{M}\mathrm{V}1}$, $\:{\mathrm{F}}_{\mathrm{G}\mathrm{R}\mathrm{M}\mathrm{Y}}$, and $\:{\mathrm{F}}_{\mathrm{G}\mathrm{H}\mathrm{A}\mathrm{P}}$ displayed coefficients below 0.20. As for the difference in correlation coefficients among $\:{\mathrm{F}}_{\mathrm{R}\mathrm{O}\mathrm{H}4}$, $\:{\mathrm{F}}_{\mathrm{R}\mathrm{O}\mathrm{H}4\mathrm{a}\mathrm{l}\mathrm{l}}$, $\:{\mathrm{F}}_{\mathrm{R}\mathrm{O}\mathrm{H}16}$, and $\:{\mathrm{F}}_{\mathrm{R}\mathrm{O}\mathrm{H}16\mathrm{a}\mathrm{l}\mathrm{l}}$, the correlation coefficients for $\:{\mathrm{F}}_{\mathrm{R}\mathrm{O}\mathrm{H}4}$ and $\:{\mathrm{F}}_{\mathrm{R}\mathrm{O}\mathrm{H}4\mathrm{a}\mathrm{l}\mathrm{l}}$ (0.85 and 0.86, respectively) were higher than those for $\:{\mathrm{F}}_{\mathrm{R}\mathrm{O}\mathrm{H}16}$ and $\:{\mathrm{F}}_{\mathrm{R}\mathrm{O}\mathrm{H}16\mathrm{a}\mathrm{l}\mathrm{l}}$ (0.77), and there were no differences in the coefficients between SNPs with MAF ≥ 0.01 and all SNPs. Table 3 shows the descriptive statistics of the inbreeding coefficients in real cattle populations. The mean values of the inbreeding coefficients showed a similar trend among the genome-based measures compared with those in G10 of the simulated population.

**Table 3:** Summary statistics for the genome-based inbreeding coefficients in the real cattle population.

Measures^a^	Mean	SD	Min	Max
F_GRMV1_	0.00	0.00	−0.18	0.34
F_GRMV2_	0.37	0.00	0.29	0.55
F_GRMY_	0.00	0.01	−0.26	0.46
F_HOM_	0.00	0.00	−0.13	0.29
F_GHAP_	0.00	0.00	−0.18	0.35
F_HBD_	0.12	0.00	0.03	0.34
F_ROH4_	0.06	0.00	0.00	0.26
F_ROH4all_	0.06	0.00	0.00	0.26
F_ROH16_	0.03	0.00	0.00	0.23
F_ROH16all_	0.03	0.00	0.00	0.24

^a^The calculations and the abbreviations of genome-based measures are shown in the main text.

**Fig. 6:**
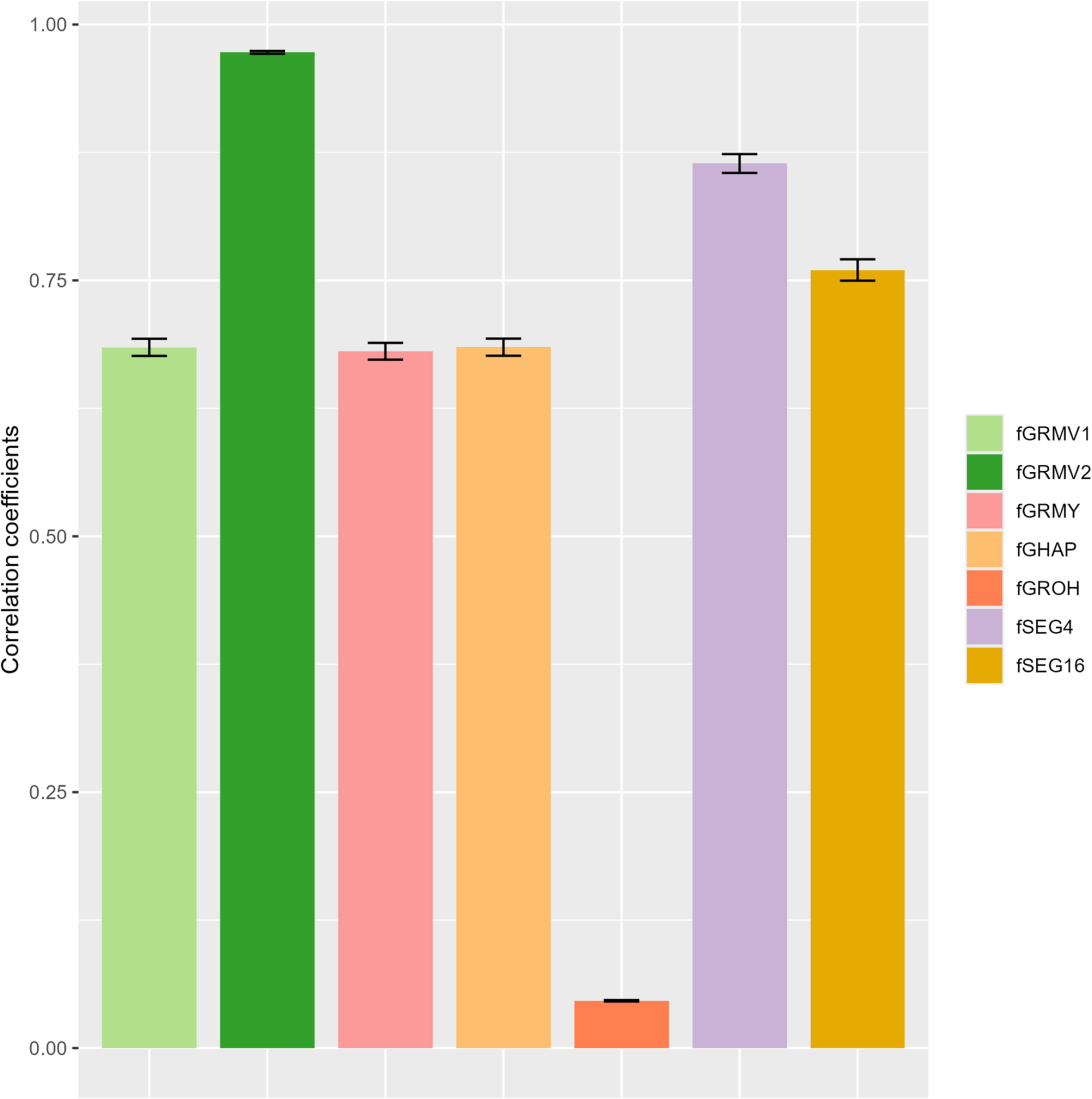
Correlation coefficients between reference and predicted values of additive relationship coefficients in the real cattle population. Correlation coefficients were calculated in the real cattle population, which was simulated by a total of 100 replicates of random extraction for the unobserved single nucleotide polymorphisms (SNPs). Mean and SD of 100 replicates were calculated and plotted. The calculations of the reference and predicted values of additive relationship coefficients are explained in the main text. The abbreviations of the genome-based measures are also explained in the main text.

The correlation coefficients between the reference and predicted values of the additive relationship coefficients in the real cattle population are shown in Fig. 6 and Supplementary Table S5. The correlation coefficients ranged from 0.05 to 0.97, with $\:{\mathrm{f}}_{\mathrm{G}\mathrm{R}\mathrm{M}\mathrm{V}2}$ and $\:{\mathrm{f}}_{\mathrm{S}\mathrm{E}\mathrm{G}4}$ exhibiting the two highest correlation coefficients (0.97 and 0.86, respectively) and $\:{\mathrm{f}}_{\mathrm{G}\mathrm{R}\mathrm{O}\mathrm{H}}$ displaying a coefficient below 0.10.

## Discussion

Management of genetic diversity in cattle breeds is important for maintaining genetic resources and avoiding inbreeding depression, and an appropriate indicator for monitoring the levels of inbreeding is necessary. There are several established genome-based measures for evaluating the levels of genomic inbreeding^41,43,46,47,50,54,55,59^. Among these genome-based measures based on SNPs at observed loci, it is crucial to identify reliable indicators that can accurately predict IBS relationships at unobserved loci. Accordingly, this study aimed to conduct a comprehensive investigation of the most appropriate indicators of inbreeding. This was achieved by comparing the predictive performance of existing indicators with the observed SNPs, under the assumption that the IBS relationship at unobserved loci is regarded as a reference value, and the IBS relationships represent IBS-based similarity scores rather than biologically defined IBD probability. A comprehensive analysis of the efficacy of genome-based measures in both simulated and real cattle populations is presented.

### Measures for monitoring genetic diversity

Management of genetic diversity plays a crucial role in maintaining genetic resources and encompasses the preservation of the genetic variability of causal variants at unknown loci for a trait that may prove valuable in the future despite currently being of no interest. The genetic selection of cattle with low enteric methane emission is a typical example^60^. Furthermore, the management of genetic variability contributes to alleviating the risk of inbreeding depression through increased homozygosity. Some recessive alleles with deleterious effects on reproductive performance, including bull conception rate and semen production traits, have been identified in genome-wide association studies (GWAS) of cattle populations^61–63^. Nevertheless, there could be still unknown loci that negatively affect reproductive traits. Consequently, it is necessary to investigate the accuracy of genome-based measures using observed SNPs to predict IBS relationships at unobserved loci as reference values for monitoring the levels of genomic inbreeding^19,20^. However, little is known about the predictive accuracy of IBS relationships in cattle. Some studies have assessed the accuracy of genome-based measures in predicting the IBD relationships in cattle^21^. If IBD relationships are used as reference values for monitoring genomic inbreeding, problems arise such as differences in results depending on which generation is assumed to be the base population. In addition, some of studies have assessed the accuracy of genome-based measures in predicting the pedigree-based inbreeding coefficient as reference values in beef^64,65^ and dairy cattle populations^66,67^, despite the potential biases of the method. Therefore, we investigated the accuracy of genome-based inbreeding coefficients and additive relationship coefficients with observed SNPs in predicting the IBS relationship with unobserved SNPs, which were assumed to be reference values.

In this study, SNP-by-SNP^41,43,46^, haplotype^48^ and homozygous segments^49,50,54,57,58^ measures were employed, which were derived from different genomic features. To obtain genome-based measures based on SNP-by-SNP and haplotype, it is necessary to have knowledge of the marker allele frequencies in the base population^41,43,48^. However, homozygous-segment-based measures do not require such information. Homozygous-segment-based measures can provide insight into historical and recent selection events by estimating short and long ROHs, respectively, which can be inferred from the extent and frequency of the ROH regions^52^. Haplotypes can also be used to describe recent IBD relationships that capture the LD between SNPs and QTL^68^. This is possible because long-shared haplotypes are likely inherited from more recent common ancestors. In contrast, SNP-by-SNP measures do not distinguish between these two time events. As these different genomic features may result in different accuracies in IBS relationship prediction, this study examined the impact of these different methods on the accuracy of IBS relationship prediction. It should be noted that our study does have certain limitations. We predict IBS relationships at unobserved loci using observed SNPs. However, this is based on indirect estimation via LD and genomic structure, rather than actual observed or biologically defined IBD probabilities.

### Inbreeding coefficients

In this study, the correlation coefficients between the reference and predicted values of the inbreeding coefficients were calculated for simulated cattle populations generated using different *N*_*e*_ values and selection criteria. The results showed that many genome-based measures had higher accuracy than pedigree-based measures and that the accuracy of pedigree-based measures decreased over generations in the selected population. This suggests that caution should be exercised when evaluating inbreeding levels by using pedigree-based measures as reference values. The decrease of the accuracy over generations in pedigree-based measures under EBV-based selection may reflect limitations in pedigree depth and resolution. Pedigree-based measures, which represent expected values for each individual, may become similar between individuals over generations. This suggests that they may deviate from the actual values of genomic inbreeding. In contrast, genome-based measures, such as $\:{\mathrm{F}}_{\mathrm{G}\mathrm{R}\mathrm{M}\mathrm{V}2}$, $\:{\mathrm{F}}_{\mathrm{H}\mathrm{O}\mathrm{M}}$, and $\:{\mathrm{F}}_{\mathrm{H}\mathrm{B}\mathrm{D}}$ demonstrated consistently high prediction accuracies across all generations and scenarios. In addition, $\:{\mathrm{F}}_{\mathrm{R}\mathrm{O}\mathrm{H}4\mathrm{a}\mathrm{l}\mathrm{l}}$ exhibited a high prediction accuracy, especially in the simulated population with selection based on EBVs.

When comparing the predicted values among different genome-based measures, it should be noted that these measures exhibit different ranges due to their definitions. For example, $\:{\mathrm{F}}_{\mathrm{G}\mathrm{R}\mathrm{M}\mathrm{V}2}$ assumes an allele frequency of 0.5 in the base population and thus takes values ranging from − 1 to 1, while $\:{\mathrm{F}}_{\mathrm{G}\mathrm{R}\mathrm{M}\mathrm{V}1}$ and $\:{\mathrm{F}}_{\mathrm{G}\mathrm{R}\mathrm{M}\mathrm{Y}}$ take values ranging from − 1 to ∞^69^. In addition, homozygous segments measures such as $\:{\mathrm{F}}_{\mathrm{R}\mathrm{O}\mathrm{H}4\mathrm{a}\mathrm{l}\mathrm{l}}$ take values ranging from 0 to 1. Consequently, comparing predicted values among genome-based measures is difficult, and the trends in the inbreeding coefficients for each generation were investigated. The results showed that the $\:{\mathrm{F}}_{\mathrm{G}\mathrm{R}\mathrm{M}\mathrm{V}2}$, $\:{\mathrm{F}}_{\mathrm{H}\mathrm{B}\mathrm{D}}$, and $\:{\mathrm{F}}_{\mathrm{R}\mathrm{O}\mathrm{H}4\mathrm{a}\mathrm{l}\mathrm{l}}$ increased over generations in all scenarios as $\:{\mathrm{F}}_{\mathrm{I}\mathrm{B}\mathrm{S}}$ increased, whereas the $\:{\mathrm{F}}_{\mathrm{H}\mathrm{O}\mathrm{M}}$ was constantly around zero over generations in all scenarios. The results of $\:{\mathrm{F}}_{\mathrm{H}\mathrm{O}\mathrm{M}}$ was due to the assumption that the expected numbers of homozygous SNPs were used to calculate $\:{\mathrm{F}}_{\mathrm{H}\mathrm{O}\mathrm{M}}$. When the expected numbers of homozygous SNPs are calculated in the current population, the mean is zero. This was the reason why the $\:{\mathrm{F}}_{\mathrm{H}\mathrm{O}\mathrm{M}}$ was consistently around zero over generations in all scenarios, and this result indicated that the $\:{\mathrm{F}}_{\mathrm{H}\mathrm{O}\mathrm{M}}$ has a potential to reflect the masking of true inbreeding trends.

The correlation coefficients between the reference and predicted values of the inbreeding coefficients were evaluated in a real cattle population, which indicated that the genome-based measures with high correlation coefficients (> 0.90) were $\:{\mathrm{F}}_{\mathrm{G}\mathrm{R}\mathrm{M}\mathrm{V}2}$, $\:{\mathrm{F}}_{\mathrm{H}\mathrm{O}\mathrm{M}}$, and $\:{\mathrm{F}}_{\mathrm{H}\mathrm{B}\mathrm{D}}$. The real cattle population used in this study was considered similar to a non-selected population, as animals that were very close relatives were excluded, and the results were similar to those observed in the simulated cattle population with random selection. Consequently, the results indicate that $\:{\mathrm{F}}_{\mathrm{G}\mathrm{R}\mathrm{M}\mathrm{V}2}$ and $\:{\mathrm{F}}_{\mathrm{H}\mathrm{B}\mathrm{D}}$ could be useful indicators with the highest prediction accuracy in both selected and non-selected populations. Additionally, $\:{\mathrm{F}}_{\mathrm{R}\mathrm{O}\mathrm{H}4\mathrm{a}\mathrm{l}\mathrm{l}}$ may be a useful indicator of high prediction accuracy in selected populations. These indicators also allow for the evaluation of changes in the levels of inbreeding over generations due to selection. Because most cattle populations are under selection for economically important traits, these three indicators are considered more effective than pedigree-based measures in evaluating the levels of inbreeding.

For SNP-by-SNP and haplotype-based measures, $\:{\mathrm{F}}_{\mathrm{G}\mathrm{R}\mathrm{M}\mathrm{V}2}$ was identified as the most accurate measure and could evaluate changes in the levels of inbreeding over generations due to selection in the present study. This is contingent on the assumption that the allele frequency utilized in the calculation was fixed at 0.5, which is the frequency observed in the base population. In contrast, this approach differs from other approaches ($\:{\mathrm{F}}_{\mathrm{G}\mathrm{R}\mathrm{M}\mathrm{V}1}$, $\:{\mathrm{F}}_{\mathrm{G}\mathrm{R}\mathrm{M}\mathrm{Y}}$, $\:{\mathrm{F}}_{\mathrm{H}\mathrm{O}\mathrm{M}}$, and $\:{\mathrm{F}}_{\mathrm{G}\mathrm{H}\mathrm{A}\mathrm{P}}$) that assume that the allele frequencies utilized in the calculation are obtained from the target population. Prediction of the inbreeding coefficient using allele frequency has been reported to be influenced by the allele frequency of the target population^69^. In this study, the accuracy of the measures with allele frequencies of the target population decreased, especially in the later generations of the selected population. Furthermore, these measures did not account for changes in the levels of inbreeding over generations due to selection. Conversely, $\:{\mathrm{F}}_{\mathrm{G}\mathrm{R}\mathrm{M}\mathrm{V}2}$ exhibits high accuracy in both simulated and real cattle populations, which is thought to be due to its fixed allele frequency. $\:{\mathrm{F}}_{\mathrm{G}\mathrm{R}\mathrm{M}\mathrm{V}2}$ is highly correlated with $\:{\mathrm{F}}_{\mathrm{P}\mathrm{E}\mathrm{D}}$ in cattle populations^70^ and has been employed to monitor genetic diversity in dairy cattle at the Animal Genomics and Improvement Laboratory of the USDA^71^. Obtaining allele frequencies for a base population is challenging because the base population is often unknown. This study indicates that even the assumption of fixing the allele frequency in a base population of 0.5, provides high prediction accuracy. Additionally, caution should be exercised when utilizing SNP-by-SNP and haplotype-based measures with allele frequencies of the target population as indicators, particularly in selected populations.

For homozygous-segment-based measures, $\:{\mathrm{F}}_{\mathrm{H}\mathrm{B}\mathrm{D}}$, which assumes that two ROH segments are IBD, was identified as the most accurate indicator of homozygous-segment-based measures in both simulated and real cattle populations. In addition, $\:{\mathrm{F}}_{\mathrm{R}\mathrm{O}\mathrm{H}4\mathrm{a}\mathrm{l}\mathrm{l}}$, which assumes that the two ROH segments are IBS, exhibits high prediction accuracy, especially in the selected population. In the present study, both measures were also effective in evaluating changes in the levels of inbreeding over generations due to selection. The information of ROH has been employed in numerous inbreeding coefficient predictions in cattle populations, with the utility of this approach having been reported^64–67^. However, appropriate parameter settings are required to estimate ROH. In this study, $\:{\mathrm{F}}_{\mathrm{H}\mathrm{B}\mathrm{D}}$ was calculated under the default setting, and the correlation coefficients for $\:{\mathrm{F}}_{\mathrm{H}\mathrm{B}\mathrm{D}}$ exceeded 0.90 in both simulated and real cattle populations. Although $\:{\mathrm{F}}_{\mathrm{H}\mathrm{B}\mathrm{D}}$ is a computationally time-consuming method, our results indicated that $\:{\mathrm{F}}_{\mathrm{H}\mathrm{B}\mathrm{D}}$ is a useful indicator for predicting IBS relationships at unobserved loci.

In this study, we calculated the inbreeding coefficients ($\:{\mathrm{F}}_{\mathrm{R}\mathrm{O}\mathrm{H}4}$, $\:{\mathrm{F}}_{\mathrm{R}\mathrm{O}\mathrm{H}4\mathrm{a}\mathrm{l}\mathrm{l}}$, $\:{\mathrm{F}}_{\mathrm{R}\mathrm{O}\mathrm{H}16}$, and $\:{\mathrm{F}}_{\mathrm{R}\mathrm{O}\mathrm{H}16}$), which required less computation time than $\:{\mathrm{F}}_{\mathrm{H}\mathrm{B}\mathrm{D}}$, using the estimated ROH under two different conditions: the length of the ROHs and the MAF condition for SNPs. Regarding the length of the ROH in the ROH estimation, the minimum length of the ROH was set to 4 and 16 Mbp in this study. This was done based on the assumption that ROH > 4 Mbp corresponds to homozygous segments of both past and recent origins, and that ROH > 16 Mbp corresponds to homozygous segments of only recent origins. Genome-based inbreeding coefficients calculated from long ROH are comparable to those derived from pedigree data in cattle population^66^. In this study, $\:{\mathrm{F}}_{\mathrm{R}\mathrm{O}\mathrm{H}4}$ and $\:{\mathrm{F}}_{\mathrm{R}\mathrm{O}\mathrm{H}4\mathrm{a}\mathrm{l}\mathrm{l}}$ had more accurate predictions than $\:{\mathrm{F}}_{\mathrm{R}\mathrm{O}\mathrm{H}16}$ and $\:{\mathrm{F}}_{\mathrm{R}\mathrm{O}\mathrm{H}16}$, respectively, suggesting that an ROH > 4 Mbp is more desirable for predicting IBS relationships at unobserved loci. However, caution should be exercised in evaluating inbreeding depression. Inbreeding depression may be negatively associated with recent inbreeding, and not only because of the total level of inbreeding^67^. Therefore, it may be better to use a long ROH when evaluating the negative effects of inbreeding on phenotypic values. Furthermore, this study did not assess the prediction accuracy under the condition that the ROHs were finely divided into shorter segments, such as 1–2 Mbp and 2–4 Mbp of ROH length. A finer classification of ROHs may be useful for predicting IBS relationships at unobserved loci. Further investigations are warranted based on these considerations.

As for the MAF condition for SNPs in the estimation of ROH, Meyermans et al.^72^ reported that extracting SNPs by the MAF condition would have a negative impact on the estimation of ROH due to the possibility of losing informative SNPs. Therefore, all SNPs and SNPs with MAF ≥ 0.01 were used to estimate ROH in this study. The prediction accuracy of homozygous-segment-based inbreeding coefficients using all SNPs ($\:{\mathrm{F}}_{\mathrm{R}\mathrm{O}\mathrm{H}4\mathrm{a}\mathrm{l}\mathrm{l}}$ and $\:{\mathrm{F}}_{\mathrm{R}\mathrm{O}\mathrm{H}16\mathrm{a}\mathrm{l}\mathrm{l}}$) was higher than those using SNPs with MAF ≥ 0.01 ($\:{\mathrm{F}}_{\mathrm{R}\mathrm{O}\mathrm{H}4}$ and $\:{\mathrm{F}}_{\mathrm{R}\mathrm{O}\mathrm{H}16}$, respectively) in simulated population. Moreover, the inbreeding coefficients using SNPs with MAF ≥ 0.01 decreased from the later generation in the simulated population with selection, while those using all SNPs increased over generations. Information on homozygous alleles, which are contained within the segment and fixed within the target population, can be considered important in the estimation of ROH. Therefore, this may be an important factor in the prediction of IBS relationships at unobserved loci. These findings indicate that the estimation of ROH should consider both the length of ROH and the MAF condition for the SNP.

### Additive relationship coefficients

In this study, many genome-based additive relationship coefficients had higher prediction accuracy than pedigree-based coefficients in the simulated population, and $\:{\mathrm{f}}_{\mathrm{G}\mathrm{R}\mathrm{M}\mathrm{V}2}$ and $\:{\mathrm{f}}_{\mathrm{S}\mathrm{E}\mathrm{G}4}$ demonstrated consistently high prediction accuracy in both simulated and real cattle populations. $\:{\mathrm{f}}_{\mathrm{G}\mathrm{R}\mathrm{M}\mathrm{V}2}$ and $\:{\mathrm{f}}_{\mathrm{S}\mathrm{E}\mathrm{G}4}$ can be classified using SNP-by-SNP and homozygous-segment-based methods, respectively. $\:{\mathrm{f}}_{\mathrm{G}\mathrm{R}\mathrm{M}\mathrm{V}2}$ are the off-diagonal elements of $\:{\mathbf{G}}_{\mathrm{V}}$ under $\:{\mathrm{p}_\mathrm{j}}=0.5$ baseline and do not discriminate alleles that are IBD or IBS at the observed loci. $\:{\mathrm{f}}_{\mathrm{S}\mathrm{E}\mathrm{G}4}$ are the predicted values of the IBD relationships of the chromosomal segments based on SNPs at the observed loci. Both genome-based measures showed high correlations with the IBS relationships at unobserved loci, but observed differences across scenarios advise cautious interpretation; we do not claim equivalence. Their prediction accuracy exhibited a trend similar to that of the inbreeding coefficients in both the simulated and real cattle populations. This study demonstrates that IBS relationships at unobserved loci between two individuals can be accurately predicted using genome-based additive relationship coefficients such as $\:{\mathrm{f}}_{\mathrm{G}\mathrm{R}\mathrm{M}\mathrm{V}2}$ and $\:{\mathrm{f}}_{\mathrm{S}\mathrm{E}\mathrm{G}4}$.

For the practical application in breeding program, additive relationship coefficients serve as indicators of genetic relatedness between individuals and play an important role, particularly in genetic conservation programs aimed at minimizing the loss of genetic variability^73^. Consequently, studies on the application of additive relationship coefficients to conservation programs must be necessary. Optimal contribution selection (OCS) uses additive relationship coefficients to minimize the loss of genetic variability while improving genetic ability^74^. Some studies on OCS using genetic relationship matrices with additive relationship coefficients as elements have been conducted^75,76^. Morales-González et al.^75^ employed VanRaden’s GRM with the allele frequencies of the base population and conducted a selection simulation, which demonstrated that it was feasible to maintain allele frequencies close to those of the base population. The results of OCS simulation using $\:{\mathrm{f}}_{\mathrm{S}\mathrm{E}\mathrm{G}}$ in a simulated pig population also demonstrated that genomic mating using $\:{\mathrm{f}}_{\mathrm{S}\mathrm{E}\mathrm{G}}$ is more efficient than traditional mating schemes^76^. The $\:{\mathrm{f}}_{\mathrm{S}\mathrm{E}\mathrm{G}}$ is an indicator that evaluates the proportion of ROH sharing between two individuals^59^, and genomic mating using $\:{\mathrm{f}}_{\mathrm{S}\mathrm{E}\mathrm{G}}$ can reduce the length of ROH segments within the offspring in the next generation. As this study focused on the prediction of IBS relationships at unobserved loci, suitable indicators for OCS were not evaluated. Future investigation should be conducted to determine the efficacy of OCS using $\:{\mathrm{f}}_{\mathrm{G}\mathrm{R}\mathrm{M}\mathrm{V}2}$ and $\:{\mathrm{f}}_{\mathrm{S}\mathrm{E}\mathrm{G}4}$ in cattle.

## Conclusion

We investigated whether genome-based inbreeding coefficients and additive relationship coefficients, using observed SNPs, could be used as indicators for predicting the probability of alleles at unobserved loci being IBS in an individual and between two individuals in both simulated and real cattle populations. The results demonstrated that the inbreeding coefficients and additive relationship coefficients based on SNP-by-SNP with an allele frequency fixed at 0.5 ($\:{\mathrm{F}}_{\mathrm{G}\mathrm{R}\mathrm{M}\mathrm{V}2}$ and $\:{\mathrm{f}}_{\mathrm{G}\mathrm{R}\mathrm{M}\mathrm{V}2}$, respectively) exhibited high prediction accuracy in both simulated and real cattle populations. Additionally, homozygous-segment-based inbreeding coefficients and additive relationship coefficients, which include $\:{\mathrm{F}}_{\mathrm{H}\mathrm{B}\mathrm{D}}$ and measures with short ROH ($\:{\mathrm{F}}_{\mathrm{R}\mathrm{O}\mathrm{H}4\mathrm{a}\mathrm{l}\mathrm{l}}$ and $\:{\mathrm{f}}_{\mathrm{S}\mathrm{E}\mathrm{G}4}$, respectively), were highly accurate measures in both simulated and real cattle populations. Furthermore, $\:{\mathrm{F}}_{\mathrm{G}\mathrm{R}\mathrm{M}\mathrm{V}2}$, $\:{\mathrm{F}}_{\mathrm{H}\mathrm{B}\mathrm{D}}$, and $\:{\mathrm{F}}_{\mathrm{R}\mathrm{O}\mathrm{H}4\mathrm{a}\mathrm{l}\mathrm{l}}$ were effective in evaluating changes in inbreeding levels over generations due to selection. Our results indicate that these indicators are more reliable than pedigree-based measures specifically for predicting IBS relationships at unobserved loci. Our results show that it is possible to predict the IBS relationships at unobserved loci by indirect estimation via LD and genomic structure using observed SNPs in cattle populations.

## Supplementary Information

Below is the link to the electronic supplementary material.

Supplementary Material 1

## Data Availability

The datasets analyzed during the current study are not publicly available due to the fact that the SNP genotype data of Japanese Black cattle is owned by the Japan Livestock Technology Association but are available from the corresponding author on reasonable request.
