# Supplementary material for: Probabilities of two alleles being identity by state at unobserved loci predicted by observed loci in cattle populations
Source: Sci Rep. 2026 Feb 5;16:7454. doi: 10.1038/s41598-026-37530-x (PMC12929693; doi:10.1038/s41598-026-37530-x)
Supplement: Supplementary file 1 — Supplementary Material 1 [file 41598_2026_37530_MOESM1_ESM.pdf]

Probabilities of two alleles being identity by state at unobserved loci predicted by observed loci in cattle populations

Rintaro Nagai[1], **Takeshi Honda**[2], **Masahiro Satoh**[1], **Yoshinobu Uemoto**[1*]

[1] *Graduate School of Agricultural Science, Tohoku University, Sendai, Miyagi 980-8572, Japan*

[2] *Food Resources Education and Research Center, Kobe University, Kasai, Hyogo 675-2103, Japan*

*Corresponding author

Journal: Scientific Reports

Supplementary Materials

Supplementary Figure S1. Average linkage disequilibrium (LD) coefficient values (r^2 values) plotted against intermarker distance in the simulated cattle population.

Supplementary Figure S2. Correlation coefficients between the identity-by-state (IBS) relationships at different numbers of single nucleotide polymorphisms (SNPs) in the simulated cattle population.

Supplementary Table S1. The details of genomic structure of the simulated cattle population.

Supplementary Table S2. Correlation coefficients between reference and predicted values of inbreeding coefficients in the simulated cattle population.

Supplementary Table S3. Correlation coefficients between reference and predicted values of additive relationship coefficients in the simulated cattle population.

Supplementary Table S4. Correlation coefficients between reference and predicted values of inbreeding coefficients in the real cattle population.

Supplementary Table S5. Correlation coefficients between reference and predicted values of additive relationship coefficients in the real cattle population.

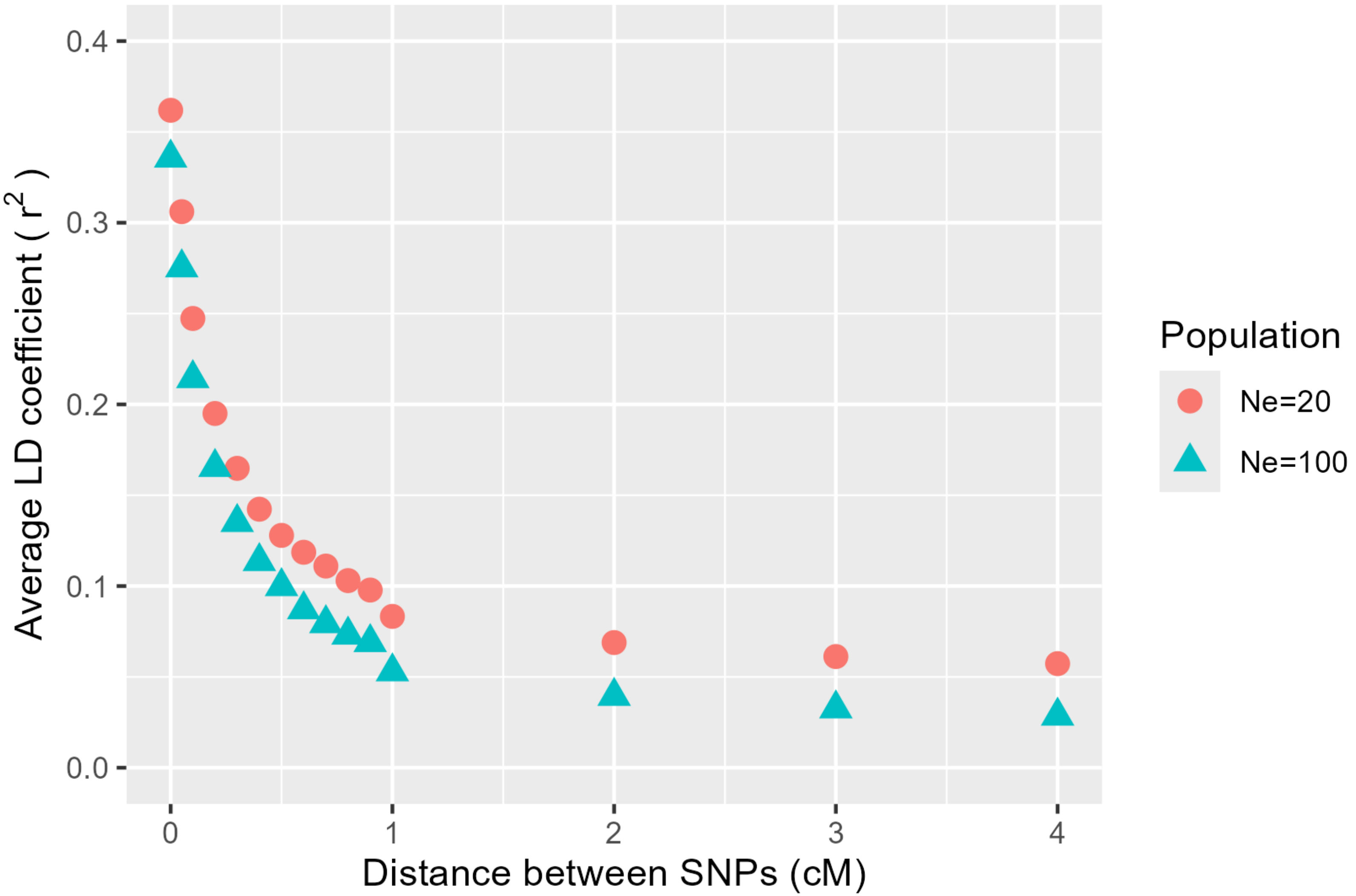

Supplementary Figure S1. Average linkage disequilibrium (LD) coefficient values (r^2 values) plotted against intermarker distance in the simulated cattle population. X-axis represents the distance between single nucleotide polymorphisms (SNPs) and Y-axis indicates the r^2 values between SNPs, which were measures of LD between any two loci in the base population and supplied by a feature of QMSim software [24]. r^2 values in two simulated populations (N_e=20 and N_e=100) were randomly extracted.

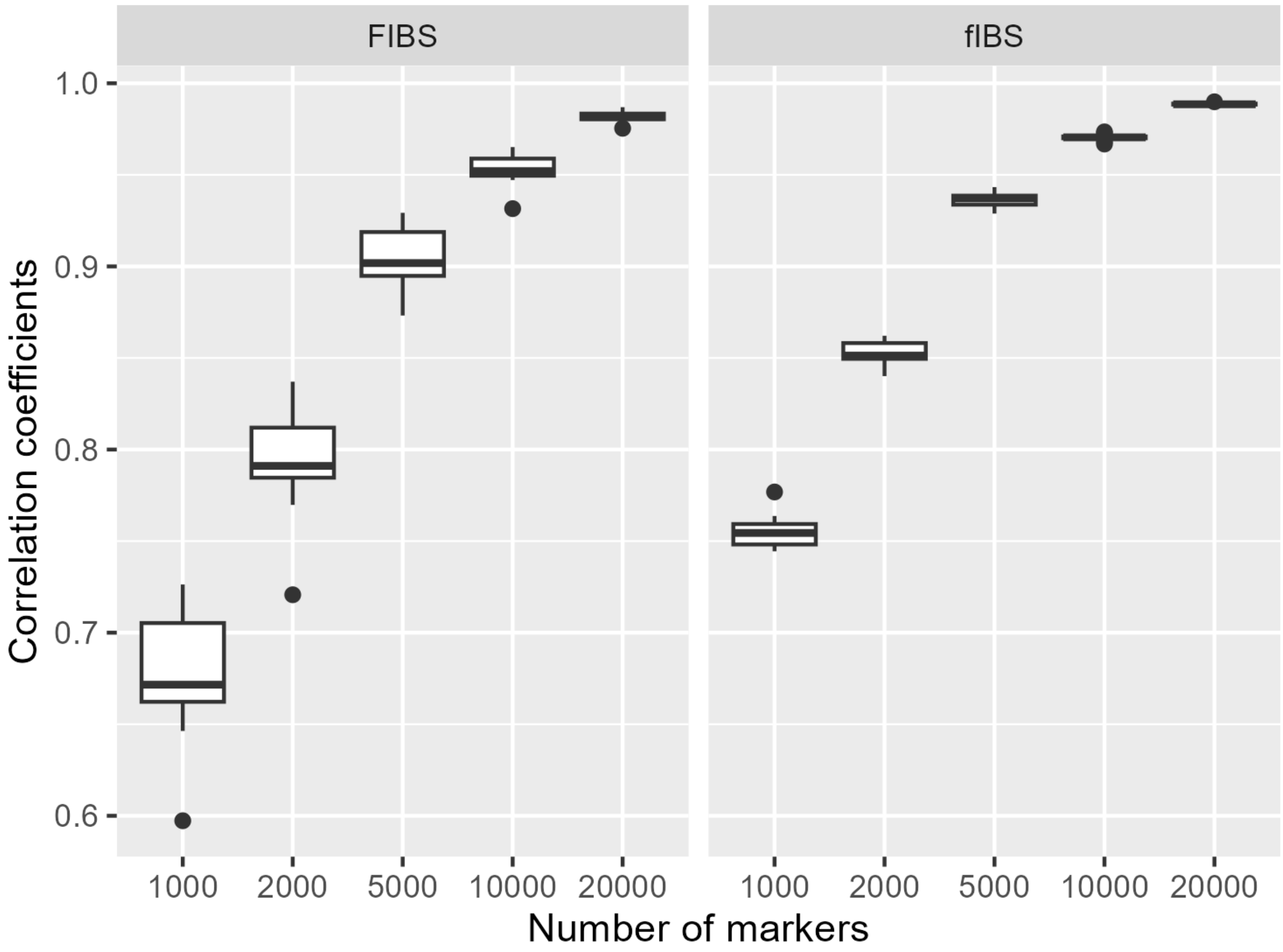

Supplementary Figure S2. Correlation coefficients between the identity-by-state (IBS) relationships at different numbers of single nucleotide polymorphisms (SNPs) in the simulated cattle population. Correlation coefficients between 50,000 SNPs and 1,000, 2,000, 5,000, 10,000, and 20,000 SNPs were calculated for both the inbreeding coefficient (FIBS) and the additive relationship coefficient (fIBS) at generation 100 in the simulated cattle population with N_e=100 and selection at random. The correlation coefficients of 20 replicates were plotted, and the calculations of FIBS and fIBS are explained in the main text.

Supplementary Table S1. The details of genomic structure of the simulated cattle population[a]

Chromosome	cM	nSNP1	nSNP2	nQTL
1	158	3,412	6,824	70
2	136	2,816	5,632	70
3	120	2,555	5,110	70
4	119	2,563	5,126	70
5	120	2,262	4,524	70
6	117	2,526	5,052	70
7	110	2,354	4,708	70
8	113	2,416	4,832	70
9	104	2,079	4,158	70
10	103	2,184	4,368	70
11	106	2,292	4,584	70
12	87	1,766	3,532	70
13	83	1,833	3,666	70
14	82	1,809	3,618	70
15	85	1,759	3,518	70
16	80	1,707	3,414	70
17	72	1,594	3,188	70
18	65	1,363	2,726	70
19	63	1,411	2,822	70
20	71	1,559	3,118	70
21	69	1,458	2,916	70
22	60	1,304	2,608	70
23	52	1,083	2,166	70
24	62	1,305	2,610	70
25	42	991	1,982	70
26	51	1,103	2,206	70
27	45	996	1,992	70
28	45	973	1,946	70
29	51	1,078	2,156	70
Total	2,471	52,551	105,102	2,030

[a]cM: Centi Morgan in each chromosome based on ARS-UCD 1.2 reference sequence assembly, nSNP1: Number of single nucleotide polymorphisms (SNPs) in the BovineSNP50 BeadChip, nSNP2: Number of SNPs allocated in the histrical population, which is twice as many SNPs as nSNP1, nQTL: Number of quantitative trait locus (QTL).

Supplementary Table S2. Correlation coefficients between reference and predicted values of inbreeding coefficients in the simulated cattle population[a]

Population	Measures	G10		G20		G50		G100	
		Mean	SD	Mean	SD	Mean	SD	Mean	SD
Ne=20_ran	F_{APED}	0.56	0.07	0.57	0.07	0.56	0.08	0.56	0.07
	F_{PED}	0.56	0.07	0.57	0.07	0.56	0.08	0.56	0.07
	F_{GRMV1}	0.80	0.03	0.79	0.03	0.76	0.04	0.73	0.04
	F_{GRMV2}	0.94	0.01	0.94	0.01	0.93	0.01	0.93	0.01
	F_{GRMY}	0.68	0.05	0.64	0.05	0.58	0.06	0.53	0.06
	F_{HOM}	0.94	0.01	0.94	0.01	0.93	0.01	0.93	0.01
	F_{GHAP}	0.79	0.04	0.77	0.03	0.74	0.04	0.72	0.05
	F_{HBD}	0.94	0.01	0.93	0.01	0.93	0.01	0.93	0.01
	F_{ROH4}	0.91	0.01	0.90	0.02	0.89	0.02	0.89	0.02
	$F_{ROH4all}$	0.91	0.01	0.90	0.02	0.89	0.02	0.89	0.02
	F_{ROH16}	0.84	0.02	0.82	0.03	0.79	0.04	0.77	0.04
	$F_{ROH16all}$	0.84	0.02	0.82	0.03	0.79	0.04	0.77	0.04
Ne=20_sel	F_{APED}	0.65	0.05	0.57	0.05	0.49	0.06	0.44	0.06
	F_{PED}	0.65	0.05	0.57	0.05	0.49	0.06	0.44	0.06
	F_{GRMV1}	0.61	0.09	0.49	0.09	0.40	0.09	0.39	0.07
	F_{GRMV2}	0.97	0.01	0.97	0.00	0.97	0.00	0.95	0.00
	F_{GRMY}	0.30	0.11	0.14	0.11	0.02	0.09	0.03	0.08
	F_{HOM}	0.97	0.01	0.97	0.00	0.97	0.00	0.95	0.00
	F_{GHAP}	0.58	0.09	0.46	0.10	0.38	0.09	0.38	0.07
	F_{HBD}	0.96	0.01	0.96	0.00	0.96	0.01	0.94	0.01
	F_{ROH4}	0.95	0.01	0.95	0.01	0.94	0.01	0.87	0.02
	$F_{ROH4all}$	0.95	0.01	0.95	0.01	0.95	0.01	0.92	0.01
	F_{ROH16}	0.90	0.01	0.89	0.01	0.81	0.02	0.68	0.03
	$F_{ROH16all}$	0.90	0.01	0.89	0.01	0.83	0.02	0.75	0.02
Ne=100_ran	F_{APED}	0.64	0.07	0.62	0.07	0.60	0.07	0.57	0.07
	F_{PED}	0.64	0.07	0.62	0.08	0.60	0.07	0.57	0.07
	F_{GRMV1}	0.81	0.03	0.80	0.04	0.78	0.04	0.75	0.04
	F_{GRMV2}	0.92	0.01	0.92	0.01	0.93	0.01	0.93	0.01
	F_{GRMY}	0.70	0.05	0.66	0.07	0.62	0.07	0.55	0.07
	F_{HOM}	0.92	0.01	0.92	0.01	0.93	0.01	0.93	0.01
	F_{GHAP}	0.80	0.04	0.79	0.05	0.77	0.05	0.73	0.05
	F_{HBD}	0.92	0.01	0.92	0.01	0.93	0.01	0.92	0.01
	F_{ROH4}	0.88	0.02	0.89	0.02	0.89	0.02	0.89	0.02
	$F_{ROH4all}$	0.88	0.02	0.89	0.02	0.89	0.02	0.89	0.02
	F_{ROH16}	0.83	0.03	0.82	0.04	0.81	0.04	0.78	0.04
	$F_{ROH16all}$	0.83	0.03	0.82	0.04	0.81	0.04	0.78	0.04
Ne=100_sel	F_{APED}	0.68	0.04	0.60	0.06	0.51	0.06	0.45	0.06
	F_{PED}	0.68	0.04	0.59	0.06	0.50	0.06	0.45	0.06
	F_{GRMV1}	0.62	0.12	0.51	0.09	0.42	0.08	0.40	0.07
	F_{GRMV2}	0.96	0.01	0.97	0.01	0.97	0.00	0.95	0.01
	F_{GRMY}	0.32	0.15	0.16	0.11	0.04	0.09	0.04	0.07
	F_{HOM}	0.96	0.01	0.97	0.01	0.97	0.00	0.95	0.01
	F_{GHAP}	0.58	0.13	0.48	0.10	0.40	0.09	0.39	0.07
	F_{HBD}	0.95	0.01	0.96	0.01	0.96	0.00	0.94	0.01
	F_{ROH4}	0.94	0.01	0.95	0.01	0.94	0.01	0.87	0.02
	$F_{ROH4all}$	0.94	0.01	0.95	0.01	0.95	0.01	0.92	0.01
	F_{ROH16}	0.91	0.02	0.89	0.02	0.83	0.02	0.69	0.04
	$F_{ROH16all}$	0.91	0.02	0.89	0.02	0.84	0.02	0.76	0.03

[a]Correlation coefficients were calculated at generation 10 (G10), 20 (G20), 50 (G50), and 100 (G100) in the simulated cattle population with four combinations of effective population size (N_e=20 and N_e=100) and selection criteria (ran: at random and sel: selection based on estimated breeding values) in each replicate. Mean and SD of 100 replicates were calculated and are shown. The calculations of the reference and predicted values of inbreeding coefficients are explained in the main text. The abbreviations of the pedigree- and genome-based measures are also explained in the main text.

Supplementary Table S3. Correlation coefficients between reference and predicted values of additive relationship coefficients in the simulated cattle population[a]

		G10		G20		G50		G100	
Population	Measures	Mean	SD	Mean	SD	Mean	SD	Mean	SD
Ne=20_ran	f_{APED}	0.78	0.01	0.79	0.01	0.78	0.01	0.77	0.01
	f_{PED}	0.78	0.01	0.78	0.01	0.78	0.01	0.77	0.01
	f_{GRMV1}	0.93	0.01	0.93	0.00	0.92	0.01	0.91	0.01
	f_{GRMV2}	0.96	0.00	0.96	0.00	0.96	0.00	0.96	0.00
	f_{GRMY}	0.93	0.01	0.93	0.00	0.92	0.01	0.91	0.01
	f_{GHAP}	0.93	0.01	0.93	0.00	0.92	0.01	0.91	0.01
	f_{GROH}	0.09	0.01	0.11	0.01	0.13	0.01	0.17	0.01
	f_{SEG4}	0.94	0.00	0.94	0.00	0.93	0.00	0.93	0.00
	f_{SEG16}	0.90	0.00	0.90	0.00	0.88	0.00	0.87	0.00
Ne=20_sel	f_{APED}	0.76	0.01	0.70	0.02	0.62	0.02	0.58	0.02
	f_{PED}	0.76	0.01	0.70	0.02	0.62	0.02	0.58	0.02
	f_{GRMV1}	0.86	0.03	0.82	0.03	0.78	0.03	0.77	0.02
	f_{GRMV2}	0.97	0.00	0.98	0.00	0.97	0.00	0.96	0.00
	f_{GRMY}	0.86	0.03	0.81	0.03	0.76	0.03	0.75	0.03
	f_{GHAP}	0.86	0.03	0.82	0.03	0.78	0.03	0.77	0.02
	f_{GROH}	0.13	0.01	0.18	0.02	0.26	0.01	0.27	0.01
	f_{SEG4}	0.96	0.00	0.96	0.00	0.95	0.00	0.90	0.01
	f_{SEG16}	0.93	0.01	0.91	0.01	0.86	0.01	0.79	0.02
Ne=100_ran	f_{APED}	0.83	0.00	0.82	0.01	0.80	0.00	0.78	0.01
	f_{PED}	0.83	0.00	0.82	0.01	0.80	0.01	0.78	0.01
	f_{GRMV1}	0.93	0.00	0.93	0.01	0.92	0.01	0.91	0.01
	f_{GRMV2}	0.96	0.00	0.96	0.00	0.96	0.00	0.96	0.00
	f_{GRMY}	0.93	0.00	0.93	0.01	0.92	0.01	0.91	0.01
	f_{GHAP}	0.93	0.00	0.93	0.01	0.93	0.01	0.91	0.01
	f_{GROH}	0.08	0.00	0.09	0.01	0.11	0.01	0.14	0.01
	f_{SEG4}	0.93	0.00	0.93	0.00	0.93	0.00	0.93	0.00
	f_{SEG16}	0.91	0.00	0.91	0.00	0.89	0.00	0.88	0.00
Ne=100_sel	f_{APED}	0.80	0.01	0.72	0.02	0.63	0.02	0.59	0.02
	f_{PED}	0.80	0.01	0.72	0.02	0.63	0.02	0.59	0.02
	f_{GRMV1}	0.87	0.04	0.83	0.03	0.78	0.03	0.78	0.02
	f_{GRMV2}	0.97	0.00	0.97	0.00	0.97	0.00	0.96	0.00
	f_{GRMY}	0.87	0.04	0.83	0.03	0.77	0.03	0.76	0.02
	f_{GHAP}	0.87	0.04	0.83	0.03	0.78	0.03	0.78	0.02
	f_{GROH}	0.11	0.01	0.17	0.02	0.25	0.01	0.27	0.01
	f_{SEG4}	0.96	0.00	0.96	0.00	0.95	0.00	0.91	0.01
	f_{SEG16}	0.93	0.01	0.92	0.01	0.86	0.01	0.80	0.01

[a]Correlation coefficients were calculated at generation 10 (G10), 20 (G20), 50 (G50), and 100 (G100) in the simulated cattle population with four combinations of effective population size (N_e=20 and N_e=100) and selection criteria (ran: at random and sel: selection based on estimated breeding values) in each replicate. Mean and SD of 100 replicates were calculated and shown. The calculations of the reference and predicted values of additive relationship coefficients are explained in the main text. The abbreviations of the pedigree- and genome-based measures are also explained in the main text.

Supplementary Table S4. Correlation coefficients between reference and predicted values of inbreeding coefficients in the real cattle population[a]

Measures	Mean	SD
F_{GRMV1}	0.20	0.02
F_{GRMV2}	0.96	0.00
F_{GRMY}	-0.11	0.01
F_{HOM}	0.96	0.00
F_{GHAP}	0.18	0.02
F_{HBD}	0.90	0.01
F_{ROH4}	0.85	0.01
$F_{ROH4all}$	0.86	0.01
F_{ROH16}	0.77	0.01
$F_{ROH16all}$	0.77	0.01

[a]Correlation coefficients were calculated in the real cattle population, which was simulated by a total of 100 replicates of random extraction for the unobserved single nucleotide polymorphisms (SNPs). Mean and SD of 100 replicates were calculated and shown. The calculations of the reference and predicted values of inbreeding coefficients are explained in the main text. The abbreviations of the genome-based measures are also explained in the main text.

Supplementary Table S5. Correlation coefficients between reference and predicted values of additive relationship coefficients in the real cattle population[a]

Measures	Mean	SD
f_{GRMV1}	0.68	0.01
f_{GRMV2}	0.97	0.00
f_{GRMY}	0.68	0.01
f_{GHAP}	0.68	0.01
f_{GROH}	0.05	0.00
f_{SEG4}	0.86	0.01
f_{SEG16}	0.76	0.01

[a]Correlation coefficients were calculated in the real cattle population, which was simulated by a total of 100 replicates of random extraction for the unobserved single nucleotide polymorphisms (SNPs). Mean and SD of 100 replicates were calculated and shown. The calculations of the reference and predicted values of additive relationship coefficients are explained in the main text. The abbreviations of the genome-based measures are also explained in the main text.
